# Prediction of Optimal Drug Schedules for Controlling Autophagy

**DOI:** 10.1038/s41598-019-38763-9

**Published:** 2019-02-05

**Authors:** Afroza Shirin, Isaac S. Klickstein, Song Feng, Yen Ting Lin, William S. Hlavacek, Francesco Sorrentino

**Affiliations:** 1 Mechanical Engineering Department, University of New Mexico, Albuquerque, NM 87131 USA; 2 Theoretical Biology and Biophysics Group, Theoretical Division and Center for Nonlinear Studies, Los Alamos National Laboratory, Los Alamos, NM 87545 USA

## Abstract

The effects of molecularly targeted drug perturbations on cellular activities and fates are difficult to predict using intuition alone because of the complex behaviors of cellular regulatory networks. An approach to overcoming this problem is to develop mathematical models for predicting drug effects. Such an approach beckons for co-development of computational methods for extracting insights useful for guiding therapy selection and optimizing drug scheduling. Here, we present and evaluate a generalizable strategy for identifying drug dosing schedules that minimize the amount of drug needed to achieve sustained suppression or elevation of an important cellular activity/process, the recycling of cytoplasmic contents through (macro)autophagy. Therapeutic targeting of autophagy is currently being evaluated in diverse clinical trials but without the benefit of a control engineering perspective. Using a nonlinear ordinary differential equation (ODE) model that accounts for activating and inhibiting influences among protein and lipid kinases that regulate autophagy (MTORC1, ULK1, AMPK and VPS34) and methods guaranteed to find locally optimal control strategies, we find optimal drug dosing schedules (open-loop controllers) for each of six classes of drugs and drug pairs. Our approach is generalizable to designing monotherapy and multi therapy drug schedules that affect different cell signaling networks of interest.

## Introduction

Although there is much current interest in using combinations of molecularly targeted drugs to improve outcomes for cancer patients^1,2^, relatively little work has been done in the area of formal therapy design, meaning therapy selection and/or scheduling driven by insights from mathematical models^3,4^. Formal approaches to therapy design are potentially useful for at least three reasons. First, all possible combinations of drugs may be difficult, if not impossible, to evaluate experimentally simply because of the large number of possible combinations. Second, an ability to extrapolate accurately beyond well-characterized scenarios with the aid of predictive models would be valuable for individualized treatment, especially in cases where molecular causes of disease are diverse and vary from patient to patient, as in many forms of cancer^5^. Third, it is often non-obvious how the immediate effects of drug perturbations propagate through a cellular regulatory network to affect cellular phenotypes and fates^6^ or how drug combinations might be deployed to avoid or delay the emergence of resistance, a common response of malignant cells to targeted therapies^7^. Predictive models promise to help identify new robust therapies.

Here, we apply mathematical modeling and optimal control methods to design drug schedules for manipulating autophagy, a stress-relieving/homeostatic cellular recycling process that, when nutrients are in limited supply, generates building blocks for protein synthesis through degradation of cytoplasmic contents^8^, such as cytotoxic protein aggregates that are too large for proteosomal degradation and damaged organelles (e.g., depolarized mitochondria). Autophagy also plays an important role in immunity^9,10^; the autophagic degradative machinery can be directed to target intracellular microbes, such as *Mycobacterium tuberculosis*, for destruction.

Cytoplasmic contents that are targeted for autophagic degradation are first trapped in double-membrane vesicles, termed autophagosomes or autophagic vesicles (AVs), and then delivered to lysosomes for digestion^11,12^. The production of AVs is controlled by an intricate regulatory network, in which three protein kinase-containing complexes are prominent: the heterotrimeric AMP-activated kinase (AMPK), which senses energy (glucose) supply through interactions with adenosine derivatives (AMP and ATP)^13,14^; MTOR complex 1 (MTORC1), which senses amino acid supply and growth factor signaling through interactions with small GTPases localized to lysosomal surfaces (Rag proteins and RHEB)^15,16^; and the ULK1 complex, which is activated by AMPK and repressed by MTORC1^17–19^. A fourth complex, which contains a lipid kinase, VPS34, also plays an important role^20,21^. Interestingly, VPS34 and MTOR are phylogenetically related: they are both members of the phosphoinositide 3-kinase (PI3K) family. Drugs with specificity for each of these kinases are available, and because of the relationship between MTOR and VPS34, drugs are also available with dual specifity for this pair of kinases^22–24^.

In cancer, and other contexts, autophagy is a double-edged sword^25^. It can protect cancer cells from stresses of the tumor environment (e.g., lack of nutrients because of defective vasculature) or induce cell death if recycling is excessive. Thus, there are potential benefits to be gained by using drugs to either upregulate autophagy (to kill malignant cells through excessive recycling) or downregulate autophagy (to kill cancer cells that rely on autophagy for survival)^26^.

To investigate how single drugs and drug pairs might be best used for these purposes, we constructed a system of nonlinear ordinary differential equations (ODE) that captures regulatory interactions between MTORC1, ULK1, AMPK, and VPS34, as well as the idealized pharmacokinetics of kinase inhibitors specific for MTORC1, ULK1, AMPK, and VPS34, such as rapamycin^27^, SBI-0206965^28^, dorsomorphin^29^, and SAR405^30^, respectively. We also considered an allosteric activator of AMPK (e.g., PF-06409577^31^) and a kinase inhibitor with dual specificity for MTORC1 and VPS34 (e.g., buparlisib^32^). Although the model is minimalist by design, it reproduces key behavioral features of earlier, more mechanistically detailed models^33,34^, such as oscillatory responses to intermediate levels of nutrient or energy stress. We then applied optimization methods implemented in the open-source ${\mathscr{P}}{\mathscr{S}}{\mathscr{O}}{\mathscr{P}}{\mathscr{T}}$ software package^35^ to find locally optimal dosing schedules that minimize the total amount of drug needed to drive the network to a desired, non-attracting operating point (corresponding to low or high AV count/turnover) and maintain it there. The dosing schedules are non-obvious, and synergistic drug pairs were predicted (drug 6 plus drug 1, 2 or 3), such as the combination of a VPS34 inhibitor and a dual specificity PI3K inhibitor, which acts on both VPS34 and MTORC1. This drug pair requires less total drug to achieve the same effect than either of the individual drugs alone and is relatively fast acting, which may be important for preventing or slowing the emergence of resistance.

The approach illustrated here differs from earlier applications of control theory concepts in the area of formal therapy design^36–40^ in that 1) the system being controlled is a cellular regulatory network, 2) the control interventions are injections (i.e., inputs) of (combinations of) molecularly targeted drugs, and 3) the control objective is manipulation of a cellular phenotype, namely the number of AVs per cell, which is related to the rate of AV turnover, with minimization of total drug used and a constraint on the maximum instantaneous drug concentration. The rationale for minimizing drug use is to avoid offtarget effects and associated toxicities. Our work is distinct from earlier studies of (non-biological) nonlinear network control^41–44^, in that our control goal is not to drive the system to an attractor (e.g., a stable steady state or limit cycle), but to an arbitrary point in phase space (i.e., the multidimensional space defined by the state variables of a system) and to then maintain the system there indefinitely. The approach is both flexible and generalizable and provides a means for computationally prioritizing drug dosing schedules for experimental evaluation.

## Results

### Model for cellular regulation of autophagy and the effects of targeted drug interventions

**Figure 1:**
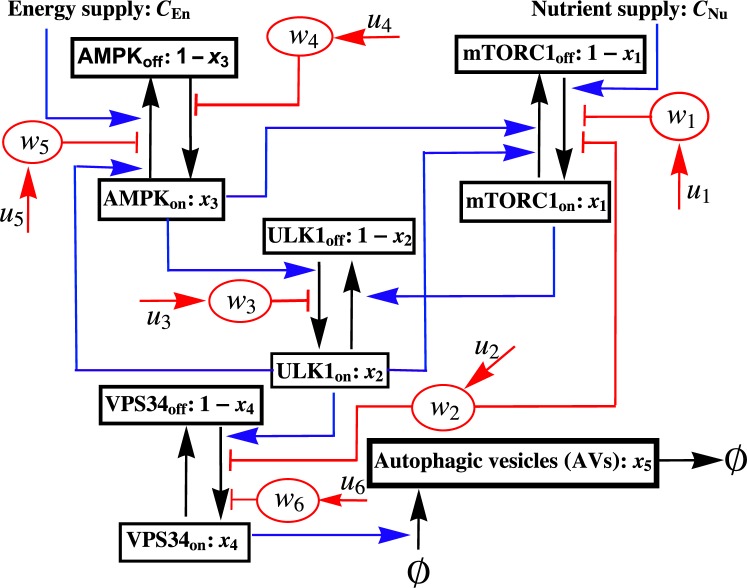
Schematic diagram of a minimalist mathematical model for regulation of autophagy and the effects of targeted drug interventions. The model accounts for two physiological inputs (energy and nutrient supply) and regulatory influences, stimulatory or inhibitory, within a network of interacting kinases. Each kinase is taken to have a constant total abundance and to be dynamically distributed between active and inactive forms. The active fractions of MTORC1, ULK1, AMPK, and VPS34 are represented by *x*_1_, *x*_2_, *x*_3_ and *x*_4_, respectively. Targeted drugs, denoted by red ovals, promote kinase inactivation or activation as indicated. Six drug types are considered: (1) a kinase inhibitor specific for MTORC1, (2) a kinase inhibitor specific for both MTORC1 and VPS34, (3) an ULK1 kinase inhibitor, (4) an allosteric activator of AMPK, (5) an AMPK kinase inhibitor, and (6) a VPS34 kinase inhibitor. The supplies of cellular energy and nutrients (*C*_En_ and *C*_Nu_), together with drug concentrations (${w}_{1},\ldots ,{w}_{6}$), determine the kinase activities of MTORC1, ULK1, AMPK, and VPS34 and thereby the rate of synthesis of autophagic vesicles (AVs). The control parameters are drug injection/input rates (${u}_{1},\ldots ,{u}_{6}$). Note that drug clearance is not indicated in this diagram but is considered in the model equations.

A prerequisite for formal therapy design is a mathematical model that captures the relevant effects of drugs of interest. Given our interest in using drugs to modify the process of (macro)autophagy, we constructed a model for regulation of the rate of synthesis of autophagic vesicles (AVs) that accounts for the enzymatic activities and interactions of four kinases that play critical roles in regulating autophagy, all of which are potential drug targets. The model further considers the effects of achievable drug interventions and idealized drug pharmacokinetics, meaning instantaneous drug injection according to a time-dependent control function and first-order clearance. The model is illustrated in Fig. 1.

The model was constructed in two steps. First, we constructed a minimalist model for physiological regulation of autophagy consistent with key features of earlier, more mechanistically detailed models^33,34^ (see “Formulation of the Model” in Supplementary Methods for details). These features include the time scale of drug-stimulated autophagy induction and the dynamic range of regulation characterized by Martin *et al*.^34^ and the qualitative system behaviors characterized by Szymańska *et al*.^33^, including a steady, low level of autophagy at low stress levels, oscillatory behavior at intermediate stress levels, and a steady, high level of autophagy at high stress levels. Simulations based on the present model—generated through numerical integration of the equations given below—and simulations based on earlier, related models^33,34^ are compared in Supplementary Fig. S1. Simulations of AV dynamics are compared to measured AV dynamics^33^ in Supplementary Fig. S2.

The model of Fig. 1 is intended to provide an idealized representation of regulation of AV synthesis in a single (average) cell in response to changes in the cellular supplies of energy and nutrients, which are treated in the model as external inputs that modulate the serine/threonine-specific protein kinase activities of AMPK and MTORC1, respectively. Thus, the model reflects regulation of AMPK activity by the cellular AMP:ATP ratio, which is affected by glucose availability, for example, and regulation of MTORC1 activity via, for example, the various amino acid-sensing regulators of Ragulator-associated heterodimeric Rag proteins, which recruit MTORC1 to lysosomes for activation in a manner that depends on their regulated guanine nucleotide binding states. The model further accounts for regulatory interactions among AMPK, MTORC1, a third serine/threonine-specific protein kinase ULK1, and a class III phosphoinositide 3-kinase (PI3K) VPS34. As noted earlier, these kinases are key regulators of autophagy, and each is a potential drug target.

In the second step of model construction, we added idealized consideration of six distinct drug interventions, which correspond to interventions achievable through use of available small-molecule compounds, such as rapamycin^27^ (an inhibitor of MTORC1 kinase activity), buparlisib^32^ (an inhibitor of PI3K-family kinases that has specificity for both MTORC1 and VPS34), SBI-206965^28^ (an inhibitor of ULK1 kinase activity), dorsomorphin^29^ (an inhibitor of AMPK kinase activity), PF-06409577^31^ (a direct activator of AMPK kinase activity), and SAR405^30^ (an inhibitor of VPS34 kinase activity). Each drug $i\in \{1,\ldots ,6\}$ (Fig. 1) is taken to be cleared via a pseudo first-order process and introduced in accordance with a specified, time-dependent injection function *u*_*i*_.

The model was formulated as a coupled system of nonlinear ordinary differential equations (ODEs):

1a $$T{\dot{x}}_{1}(t)=(1-{x}_{1}){C}_{{\rm{Nu}}}H({w}_{1})H({w}_{2})-{x}_{1}{h}_{12}({x}_{2}){h}_{13}({x}_{3}),$$

1b $$T{\dot{x}}_{2}(t)=(1-{x}_{2}){h}_{23}({x}_{3})H({w}_{3})-{x}_{2}{h}_{21}({x}_{1}),$$

1c $$T{\dot{x}}_{3}(t)=(1-{x}_{3}){k}_{1}H({w}_{4})-{C}_{{\rm{En}}}{x}_{2}{x}_{3}H({w}_{5}),$$

1d $$T{\dot{x}}_{4}(t)=(1-{x}_{4}){h}_{42}({x}_{2})H({w}_{2})H({w}_{6})-{k}_{2}{x}_{4},$$

1e $$T{\dot{x}}_{5}(t)={k}_{3}{x}_{4}-{k}_{4}{x}_{5},$$

1f $$T{\dot{w}}_{i}(t)={b}_{i}{u}_{i}(t)-{\delta }_{i}{w}_{i}(t),\,i=1,\ldots ,6.$$

In these equations, *t* is time (in min) and *T* is a timescale, which we specify as 1.0 min. The variable *x*_1_ represents the fraction of MTORC1 that is active, the variable *x*_2_ represents the fraction of ULK1 that is active, the variable *x*_3_ represents the fraction of AMPK that is active, the variable *x*_4_ represents the fraction of VPS34 that is active, and the variable *x*_5_ represents the AV count or number of AVs per cell (on a continuum scale). Thus, *x*_*i*_ always lies somewhere in the interval $[0,1]$ for $i=1,\ldots ,4$. The AV count is bounded $0\le {x}_{5}\le {k}_{3}/{k}_{4}$ because ${x}_{5}(t)=0$ implies ${\dot{x}}_{5}(t)\ge 0$ and ${x}_{5}(t)={k}_{3}/{k}_{4}$ implies ${\dot{x}}_{5}\le 0$ (by the previously stated bound on *x*_4_(*t*)). The variables ${w}_{1},\ldots ,{w}_{6}$ represent the dimensionless concentrations of drugs 1–6. Thus, ${w}_{i}\ge 0$ for each *i*. The non-dimensional parameters *C*_En_ and *C*_Nu_ are condition-dependent constants that define the supplies of energy and nutrients. An increase in energy supply is taken to positively influence the rate of deactivation of AMPK, and an increase in nutrient supply is taken to positively influence the rate of activation of MTORC1. The non-dimensional parameters *k*_1_ and *k*_2_ influence the rate of activation of AMPK and the rate of deactivation of VPS34, respectively. The non-dimensional parameter *k*_3_ is the maximal rate of VPS34-dependent synthesis of AVs, and the non-dimensional parameter *k*_4_ is the rate constant for clearance of AVs. Taking the rate of AV synthesis to be proportional to VPS34 activity is consistent with the model of Martin *et al*.^34^, as is (pseudo) first-order clearance of AVs. The non-dimensional parameters ${\delta }_{1},\ldots ,{\delta }_{6}$ are rate constants for clearance of drugs 1–6. Each *h*_*ji*_(*x*_*i*_) is a non-dimensional Hill function that has the following form:

2 $${h}_{ji}({x}_{i})={r}_{b,ji}+({r}_{m,ji}-{r}_{b,ji})\frac{{x}_{i}^{{n}_{ji}}}{{x}_{i}^{{n}_{ji}}+{\theta }_{ji}^{{n}_{ji}}}$$

where *n*_*ji*_ (the Hill coefficient), *r*_*b*,*ji*_, *r*_*m*,*ji*_ and *θ*_*ji*_ are non-negative constants. The *h* functions account for regulatory influences among the four kinases considered in the model; the influences considered are the same as those considered in the model of Szymańska *et al*.^33^ (cf. Fig. 1 and Figs 1 and 2 in ref.^33^). Each *H*(*w*_*i*_) is a non-dimensional Hill function that has the following form:

3 $$H({w}_{i})={r}_{m}-({r}_{m}-{r}_{b})\frac{{w}_{i}^{n}}{{w}_{i}^{n}+{\theta }^{n}}$$

where *n* (the Hill coefficient), *r*_*b*_, *r*_*m*_ and *θ* are non-negative constants. The *H* functions account for drug effects on kinase activities. The parameters *b*_*i*_ ($i=1,\ldots ,6$) in Eq. (1f) are Boolean variables introduced for convenience, for the purpose of defining allowable drug combinations. Recall that the *u*_*i*_ terms represent drug injection/input functions, which will be determined by solving an optimal control problem (described in the following section).

Parameter settings are summarized in Supplementary Tables S1 and S2. Each *δ* parameter was assigned a value consistent with a known drug half-life^31,45–49^ (Supplementary Table S2). Other parameters were assigned values that allow the model to reproduce the qualitative signaling behaviors of the AMPK-MTORC1-ULK1 triad characterized in the theoretical study of Szymańska *et al*.^33^ and to reproduce the timescale of autophagy induction and the range of regulation quantified experimentally in the study of Martin *et al*.^34^. According to Szymańska *et al*.^33^, at low levels of energy/nutrient stress, ULK1 activity, which can be expected to correlate with autophagic flux and AV count, is steady and low; at intermediate levels of stress, ULK1 activity is oscillatory; and at high levels of stress, ULK1 activity is steady and high. As noted earlier, in Supplementary Fig. S1, we compare simulations based on Eq. (1) with simulations based on models of Szymańska *et al*.^33^ and Martin *et al*.^34^, and in Supplementary Fig. S2, we compare simulations of AV dynamics based on Eq. (1) with experimental measurements of AV dynamics reported by Martin *et al*.^34^. Parameter settings are further explained in Supplementary Methods. In Supplementary Methods, we also elaborate on how earlier models^33,34^ guided our formulation of Eq. (1) and how these models differ from Eq. (1).

**Figure 2:**
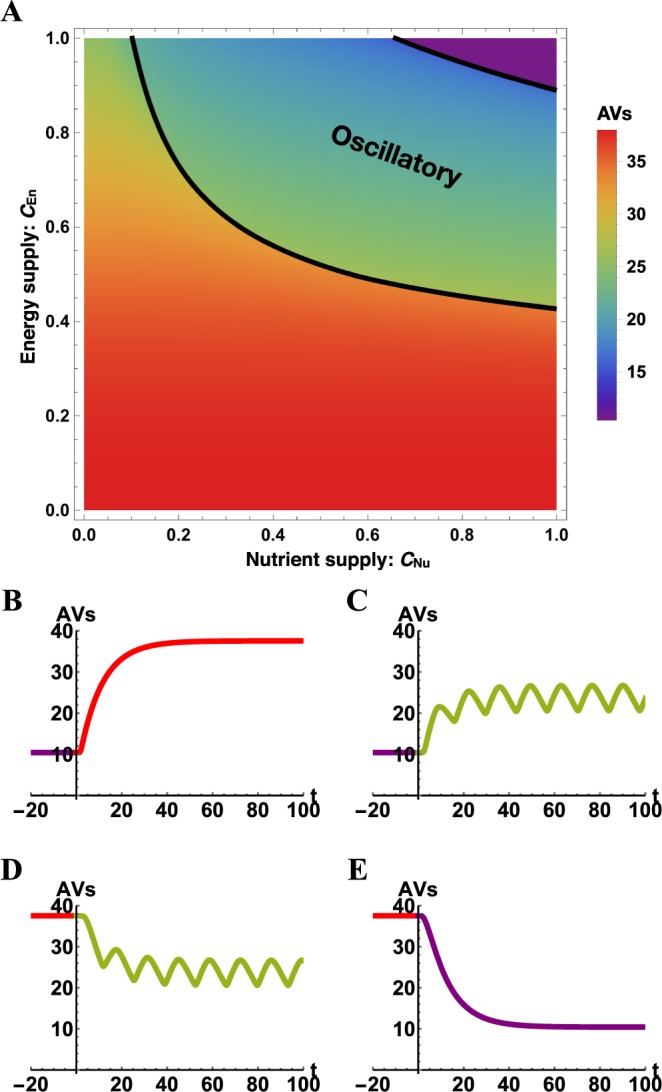
Predicted dependence of AV count on energy and nutrient supplies according to the model for autophagy regulation (Eq. (1)). (**A**) Long-time behavior. In this panel, the stationary or time-averaged value of *x*_5_(*t*) for constant supplies of energy and nutrients as $t\to \infty$ is indicated by color over the full ranges of the two physiological inputs of the model: energy supply (*C*_En_) and nutrient supply (*C*_Nu_). It should be noted that we take the most extreme energy/nutrient starvation conditions to correspond to ${C}_{{\rm{En}}}={C}_{{\rm{Nu}}}=0$, and we take the most extreme energy/nutrient replete conditions to correspond to ${C}_{{\rm{En}}}={C}_{{\rm{Nu}}}=1$. The solid black curves delimit the regions where long-time behavior of *x*_5_ is oscillatory or not. If behavior is oscillatory, the time-averaged value of *x*_5_ is reported; otherwise, the stationary value is reported. A bifurcation analysis indicates that long-time behavior is characterized by a stable fixed point, the coexistence of a stable fixed point *and* a stable limit cycle, or a stable limit cycle. The region labeled ‘oscillatory’ indicates the conditions for which a stable limit cycle exists; however, this diagram is not intended to provide a full characterization of the possible qualitative behaviors and bifurcations of Eq. (1). As indicated by the color bar, the (average) AV count varies over a range of roughly 2 to 37 vesicles per cell. (**B**–**E**) Transient behavior. Each of these plots shows *x*_5_ as a function of time *t* after a coordinated change in energy and nutrient supplies. The plot in panel *B* shows the predicted response to a steep, step increase in stress level, i.e., a change in conditions from ${C}_{{\rm{En}}}={C}_{{\rm{Nu}}}=1$ to 0.2. The plot in panel (C) shows the predicted response to a moderate, step increase in stress level, i.e., a change in conditions from ${C}_{{\rm{En}}}={C}_{{\rm{Nu}}}=1$ to 0.6. The plot in panel (D) shows the predicted response to a moderate, step decrease in stress level, i.e., a change in conditions from ${C}_{{\rm{En}}}={C}_{{\rm{Nu}}}=0.2$ to 0.6. The plot in panel *E* shows the predicted response to a steep, step decrease in stress level, i.e., a change in conditions from ${C}_{{\rm{En}}}={C}_{{\rm{Nu}}}=0.2$ to 1.

Model-predicted physiological regulation of autophagy, by energy and nutrients, is summarized in Fig. 2. Figure 2A shows how qualitative long-time behavior depends on the supplies of energy and nutrients, when these supplies are maintained at constant levels and in the absence of external control inputs $({u}_{i}=0,\,i=1,\ldots ,6)$. Figure 2B–E show time courses of autophagy induction or repression triggered by different energy/nutrient changes. All together, these plots show that model predictions of responses to physiological perturbations (i.e., changes in *C*_En_ and *C*_Nu_) are consistent with expectations based on the studies of Martin *et al*.^34^ and Szymańska *et al*.^33^.

**Figure 3:**
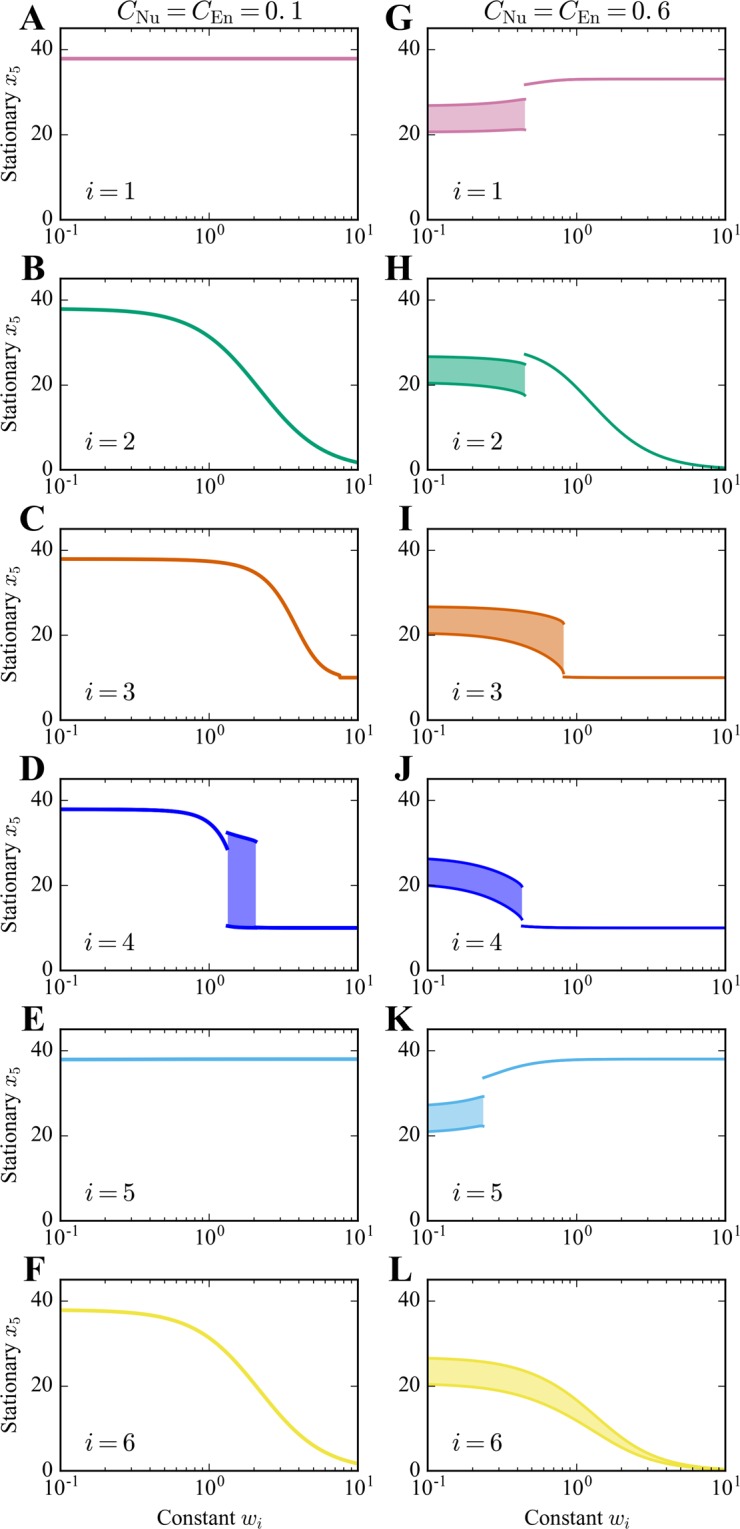
Predicted dependence of AV count (*x*_5_) on drug dose according to Eq. (1). In each panel, we show the long-time effects of monotherapy with drug $i\in \{1,\ldots ,6\}$; the drug considered in each panel is maintained at the constant (dimensionless) concentration indicated on the horizontal axis. Drugs 1–6 are considered from top to bottom. Responses to drugs depend on the supplies of energy and nutrients. The left panels (A–F) correspond to conditions for which ${C}_{{\rm{Nu}}}={C}_{{\rm{En}}}=0.1$ (severe energy/nutrient stress), and the right panels (G–L) correspond to conditions for which ${C}_{{\rm{Nu}}}={C}_{{\rm{En}}}=0.6$ (moderate energy/nutrient stress). The long-time behavior of *x*_5_ under the influence of monotherapy can be stationary (with a stable fixed point) or oscillatory (with a stable limit cycle). The shaded regions indicate where there is oscillatory behavior. At a given drug dose, the top and bottom bounds of a shaded region delimit the envelope of oscillations (i.e., the maximum and minimum values of *x*_5_).

Dose-response curves predicted by the model for single-drug, constant-concentration perturbations are shown in Fig. 3. As can be seen, with increasing dosage, drugs 1 and 5 tend to increase the number of AVs per cell, whereas the other drugs tend to decrease the number of AVs per cell. These results are consistent with negative regulation of autophagy by MTORC1 and positive regulation of autophagy by ULK1, AMPK, and VPS34. As is the case for some physiological conditions (Fig. 2), AV count oscillates at some of the drug doses, depending on the supplies of energy and nutrients. All together, the plots shown in Fig. 3 indicate that responses to single-drug, constant-concentration perturbations are consistent with accepted regulatory influences of MTORC1, ULK1, AMPK and VPS34 on autophagy.

As can be seen in Fig. 3, the ability of each drug *i* to influence *x*_5_ depends on the supplies of energy and nutrients, meaning the values of *C*_En_ and *C*_Nu_ (cf. the left and right panels in each row). In this figure, two energy/nutrient conditions are considered (${C}_{{\rm{En}}}={C}_{{\rm{Nu}}}=0.1$ and 0.6); additional conditions are considered in Supplementary Figs S3 and S4. Taken together, these results define the condition-dependent ranges over which *x*_5_ can be feasibly controlled by each drug *i*.

### Therapy design as an optimal control problem

To design optimal therapies, we must first introduce design goals. Below, we introduce a series of goals/constraints that we will require optimal therapies to satisfy. However, let us first introduce notation useful for referring to therapies. We will refer to the set of six available drugs, or more precisely, drug types, as ${\mathscr{D}}=\{1,\ldots ,6\}$, and we will refer to a therapy involving *k* drugs chosen from ${\mathscr{D}}$ as ${{\mathscr{T}}}_{k}$, where

4 $${{\mathscr{T}}}_{k}\subseteq {\mathscr{D}}\,{\rm{s}}.{\rm{t}}.\,|{{\mathscr{T}}}_{k}|=k.$$

Thus, for example, we will use ${{\mathscr{T}}}_{1}$ to refer to a monotherapy, and ${{\mathscr{T}}}_{2}$ to refer to a dual therapy. There are six possible monotherapies and, in general, ${C}_{k}^{6}$ distinct therapies that combine *k* of the six drugs. Here, we will focus on monotherapies and dual therapies, leaving the evaluation of higher-order combination therapies for future work. As a simplification, we will assume that drugs used together in a combination do not interact. Thus, for example, for dual therapy with drugs 2 and 6 (Fig. 1), we consider these drugs to bind/inhibit VPS34 independently (i.e., non-competitively).

Our first, and most important, therapy design goal can be described (somewhat informally) as follows. Starting from a stationary (or recurrent) state at time $t=0$, we wish to use drug injections (i.e., drug inputs) according to a schedule defined by $u(t)=({u}_{1}(t),\ldots ,{u}_{6}(t))$ to eventually maintain, after a transient of duration *t*_0_, the number of AVs in an average cell, *x*_5_, near (to within a tolerance *ε*) a specified target level, ${x}_{5}^{f}$, for a period of at least *t*_*f*_ − *t*_0_ (${t}_{f} > {t}_{0} > 0$), thereby achieving sustained control of the level of autophagic degradative flux in a cell, which is given by *k*_4_*x*_5_ according to Eq. (1). In our analyses, we will consider *t*_0_ = 120 min and *t*_*f*_ = 240 min because these times are longer than typical transients (Fig. 2B–E).

A second therapy design goal of interest is minimization of the total amount of drug used, which is motivated by a desire to avoid drug toxicity arising from dose-dependent offtarget effects. In the optimal control literature, a problem entailing this type of constraint is called a *minimum fuel* problem^50,51^. The constraint can be expressed mathematically as follows:

5 $$\mathop{{\rm{\min }}}\limits_{\begin{array}{c}{u}_{i}(t),\\ i\in {{\mathscr{T}}}_{k}\end{array}}\,J\{{u}_{i}\}:\,=\sum _{i\in {{\mathscr{T}}}_{k}}\,{\int }_{0}^{{t}_{f}}\,{u}_{i}(t)\,{\rm{d}}t$$

where ${u}_{i}(t)\ge 0$ for $i=1,\ldots ,6$. As a simplification, we are considering an objective functional *J*{*u*_*i*_} that treats the different drugs equally, i.e., the sum in Eq. (5) is unweighted. With this approach, we are assuming that the different drugs of interest have equivalent toxicities. If drugs are known to have different toxicities, this assumption can be lifted simply by introducing weights to capture the toxicity differences, with greater weight assigned for greater toxicity. Indeed, arbitrary modifications of the form of the objective functional *J*{*u*_*i*_} would be feasible if such modifications are needed to capture problem-specific constraints on drug dosing.

A third design goal is to disallow the instantaneous concentration of any drug *i*, *w*_*i*_(*t*), from ever rising above a threshold ${w}_{i}^{{\rm{\max }}}$. The rationale for this constraint is again related to a desire to eliminate or minimize dose-dependent drug toxicity. In other words, we are assuming that a drug *i* is tolerable so long as its concentration *w*_*i*_ is below a toxicity threshold ${w}_{i}^{{\rm{\max }}}$. In our analyses, we set the toxicity threshold of a drug as a factor (>1) times its EC_50_ dosage, which we define as the concentration of the drug at which its effect on *x*_5_, negative or positive, is half maximal (see Eqs (2) and (3)).

We are now prepared to formulate the problem of (combination) therapy design as a constrained, optimal control problem. The problem, for a given ${{\mathscr{T}}}_{k}$ (Eq. (4)), is to find a drug schedule *u*(*t*) that minimizes the objective functional defined in Eq. (5) and that also satisfies the following constraints:

6a $$\dot{{\bf{X}}}(t)={\bf{f}}({\bf{X}}(t),{\bf{u}}(t)),\,0\le t\le {t}_{f},$$

6b $${b}_{i}=\{\begin{array}{ll}1, & {\rm{if}}\,i\in {{\mathscr{T}}}_{k},\\ 0, & {\rm{otherwise}},\end{array}$$

6c $${x}_{5}^{f}-\varepsilon \le {x}_{5}(t)\le {x}_{5}^{f}+\varepsilon ,\,{t}_{0}\le t\le {t}_{f},$$

6d $$0\le {w}_{i}(t)\le {w}_{i}^{{\rm{\max }}},\,i=1,\ldots ,6,$$

6e $$0\le {u}_{i}(t),\,i=1,\ldots ,6,$$

6f $${\bf{X}}(0)=[{\bf{x}}(0),{\bf{w}}(0)]\equiv [{{\bf{x}}}_{0},{\bf{0}}].$$

Here, **X**(*t*) is defined as $[{\bf{x}}(t),{\bf{w}}(t)]$, where ${\bf{x}}(t)=({x}_{1}(t),\ldots ,{x}_{5}(t))$ and ${\bf{w}}(t)=({w}_{1}(t),\ldots ,{w}_{6}(t))$, and ${\bf{f}}({\bf{X}}(t),{\bf{u}}(t))$ is the vector field of Eq. (1). The initial condition ${{\bf{X}}}_{0}={\bf{X}}(0)$ is taken to be a stationary (or recurrent) state of Eq. (1) where supplies of energy and nutrients are constant (i.e., *C*_En_ and *C*_Nu_ are fixed) and drugs are absent (i.e., **u**(*t*) = 0). With this formulation, it should be noted that we are attempting to drive the system variable *x*_5_ to a specified final value ${x}_{5}^{f}$ (to within a tolerance *ε*), but we are making no attempt to control the other system variables *x*_1_, *x*_2_, *x*_3_, and *x*_4_. This approach is called target control^52,53^. In all of our analyses, we set $\varepsilon =1$.

A useful measure of the amount of ‘fuel’ used to achieve drug control of autophagy is the total dosage of drug *i* used up to time *t* during a therapy ${{\mathscr{T}}}_{k}$, which we denote as ${r}_{i,k}^{\ast }(t)$. This quantity is calculated using

7 $${r}_{i,k}^{\ast }(t)={\int }_{0}^{t}\,{u}_{i}^{\ast }(\tau )d\tau ,$$

where ${u}_{i}^{\ast }(t)$ for $i\in {{\mathscr{T}}}_{k}$ is the solution of the nonlinear optimal control problem defined by Eqs (5) and (6).

### Optimal monotherapies

We will illustrate generic features of solutions to the nonlinear optimal control problem defined by Eqs (5) and (6) by focusing on a particular (severe) energy/nutrient stress condition (i.e., the condition where ${C}_{{\rm{Nu}}}={C}_{{\rm{En}}}=0.1$). For this condition, the system represented by Eq. (1) has a near maximal, steady-state AV count of approximately 37 per cell (i.e., ${x}_{5}\approx 37$). Let us focus for the moment on monotherapy with drug 4 (an AMPK inhibitor) to downregulate the number of AVs to a target level of 10 per cell (i.e., ${x}_{5}^{f}=10$) over the time period between ${t}_{0}=120\,{\rm{\min }}$ and ${t}_{f}=240\,{\rm{\min }}$ from an unperturbed steady state (i.e., dynamics with *u*_*i*_ = 0) at *t* = 0.

**Figure 4:**
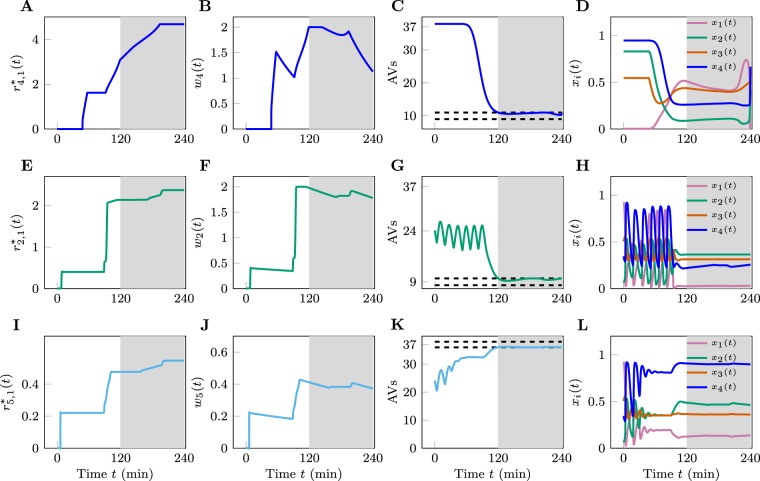
Best performing monotherapies. (**A**–**D**) Panels (A–D) are from a numerical experiment for which we set ${C}_{{\rm{Nu}}}={C}_{{\rm{En}}}=0.1$ and attempt to use drug 4 to downregulate the AV count. (*E*–*H*) Panels *E*–*H* from a numerical experiment for which we set ${C}_{{\rm{Nu}}}={C}_{{\rm{En}}}=0.6$ and attempt to use drug 2 to downregulate the AV count. (**I**–**L**) Panels (I–L) are from a numerical experiment for which we set ${C}_{{\rm{Nu}}}={C}_{{\rm{En}}}=0.6$ and attempt to use drug 5 to upregulate the AV count. The plots in the first column are cumulative drug dosages for the monotherapies considered. The plots in the second column are the drug concentrations. The plots in the third column show *x*_5_(*t*) and the plots in the fourth, or rightmost, column show *x*_1_(*t*), *x*_2_(*t*), *x*_3_(*t*), and *x*_4_(*t*) that we are making no attempt to control. In all simulations, the upper bound on the allowable concentration of drug *i*, ${w}_{i}^{{\rm{\max }}}$, was set at 2. For panels (A–H), the target AV count was 10 (i.e., ${x}_{5}^{f}=10$). For panels (I–L), the target AV count was 37 (i.e., ${x}_{5}^{f}=37$). The white region corresponds to the time interval [*t*_0_, *t*_*f*_] when we either upregulate or downregulate the AV count. The shaded region corresponds to the time interval [*t*_0_, *t*_*f*_] when the AV count is maintained within the interval ${x}_{5}^{f}\pm \varepsilon$.

We solved the optimal control problem using the approach outlined in the Methods section and described in more detail in “Pseudo-Spectral Optimal Control” in Supplementary Methods. The solution, represented by the optimal cumulative dosage of drug 4 (i.e., ${r}_{4,1}^{\ast }(t)$) (Eq. (7)), is presented in Fig. 4A. The optimal solution exhibits several generic features of the system’s dynamics, regardless of its parameterization. First, the computation suggests an optimal earliest time to apply the drug. In this particular example, this time is $t\lesssim 60\,{\rm{\min }}$. The difference between the target time *t*_0_ and the earliest time to apply the drug quantitatively measures the speed of action of the drug. Secondly, the function ${r}_{4,1}^{\ast }(t)$ exhibits a staircase behavior, indicating that the optimal strategy of drug administration for this particular problem is to intermittently inject a specific dosage of drug into the system at specific times. Mathematically, this is due to the fact that the objective functional (Eq. (5)) is a linear combination of the *L*^1^ norm of the injection/input rate *u*_*i*_’s—see Sections 5.5 and 5.6 in Kirk^50^.

Figure 4B depicts how the drug concentration *w*_4_(*t*) evolves subject to the optimal protocol ${u}_{4}^{\ast }(t)$. We observe surges of *w*_4_(*t*) in response to the drug being applied to the system in large quantities over small time intervals, and slow decays in between applications of the drug (caused by the natural decay of the drug concentration in the absence of external drug inputs dictated by *δ*_*i*_). As a consequence, the optimal solution is to inject a relatively large dose of a drug periodically, and to continuously supply small amounts of that drug to replenish drug cleared from the system to stably maintain autophagic flux (i.e., constant AV count and constant degradative flux, which we take to be proportional to the AV count).

Figure 4C illustrates the time evolution of *x*_5_ (AV count) subject to the optimal drug administration protocol. As can be seen, for $t\ge 120\,{\rm{\min }}$, *x*_5_ is maintained within the desired interval ${x}_{{5}_{f}}\pm \varepsilon =10\pm 1$. The time evolution of the non-target variables *x*_1_, *x*_2_, *x*_3_ and *x*_4_ (i.e., the activities of the regulatory kinases) are presented in Fig. 4D. Together, Fig. 4C,D provide a full representation of the time evolution of the system represented by Eq. (1) (the target and non-target variables) under the influence of the optimal drug administration schedule. Because our procedure to find the optimal solution to the nonlinear optimal control problem is numerical, we have verified that the optimal control solution satisfies the necessary conditions that it must satisfy for optimality. See “Pseudo-Spectral Optimal Control” in Supplementary Methods for details.

Given that cancer cells may be killed by using drugs to either elevate or suppress autophagy^26^, we will now consider optimal control solutions that either upregulate or downregulate autophagic flux by using a single drug. We will identify the drugs which can perturb and maintain the system near the target AV count. Perhaps more importantly, our analysis will deliver optimal protocols which include the precise times to inject the drugs, whose dosages are also tightly controlled to minimize the total quantities of drugs that are supplied.

Let us consider the case of intermediate energy/nutrient stress before treatment (i.e., the condition corresponding to ${C}_{{\rm{Nu}}}={C}_{{\rm{En}}}=0.6$; see Fig. 2), for which the system exhibits oscillations in the range $[20,27]$ without treatments. For this scenario, our goal is to either downregulate the number of AVs to ${x}_{5}^{f}\approx 9$ (shown in Fig. 4E–H) or to upregulate the AVs to ${x}_{5}^{f}\approx 37$ (shown in Fig. 4I–L). We have performed extensive numerical solutions of the monotherapy optimal control problem with various settings of the parameters ${w}_{i}^{{\rm{\max }}}$, *t*_0_, *t*_*f*_ and ${x}_{5}^{f}$. We set the control window in the interval between ${t}_{0}=120\,{\rm{\min }}$ and ${t}_{f}=240\,{\rm{\min }}$ and imposed a constraint on each drug concentration *w*_*i*_, requiring it not to exceed ${w}^{{\rm{\max }}}=4\times {{\rm{EC}}}_{50}$.

We found drug 2 to be best suited for downregulation for two reasons. First, drug 2 is able to drive *x*_5_ nearly to zero (in contrast with the case for drug 3 or 4). See Fig. 2B,H and compare with Fig. 2C,D,I,J. Second, drug 2 (in contrast with drug 6) is able to overcome the autonomous oscillatory behavior in *x*_5_. In the analysis summarized in Supplementary Fig. S7, we found that drug 6 cannot eliminate oscillatory behavior; thus, it is incapable of maintaining a low, steady AV level. Drug 6 becomes viable if we remove the lower bound from the constraint of Eq. (6c). Without the lower bound, oscillations in *x*_5_ are permitted. We choose to keep the constraint of Eq. (6c) as written to avoid oscillatory solutions because, depending on period and amplitude, oscillations in *x*_5_ may allow for autophagy-addicted cells to survive periods of relatively low autophagy by thriving during periods of relatively high autophagy. In the other direction (i.e., drug-induced upregulation of autophagy), it is only possible to use drug 5 to upregulate autophagy to the target value ${x}_{5}^{f}=37$ (Fig. 3). Figures 4E–H and I–L illustrate the optimal solutions using drugs 2 and 5 to downregulate and upregulate autophagy, respectively.

Although the selection of a single drug to achieve a given qualitative change in *x*_5_ is intuitive, especially given the results of Fig. 3, optimization of drug scheduling (Fig. 4) delivers better solutions in the sense that the total dosage applied to achieve the same effect (compared to constant input) is lower (minimized). Furthermore, the generic staircase-like solutions for ${r}_{i,k}^{\ast }$ illustrated in Fig. 4 persist for all the parameter sets we have tested (see below), indicating that variable, tightly controlled dosages should be injected into the system at controlled times. Given a particular type of drug, the central result of our optimal control analysis is to provide injection/input times and the amounts of drugs to be injected/added.

### Optimal combination therapies

**Figure 5:**
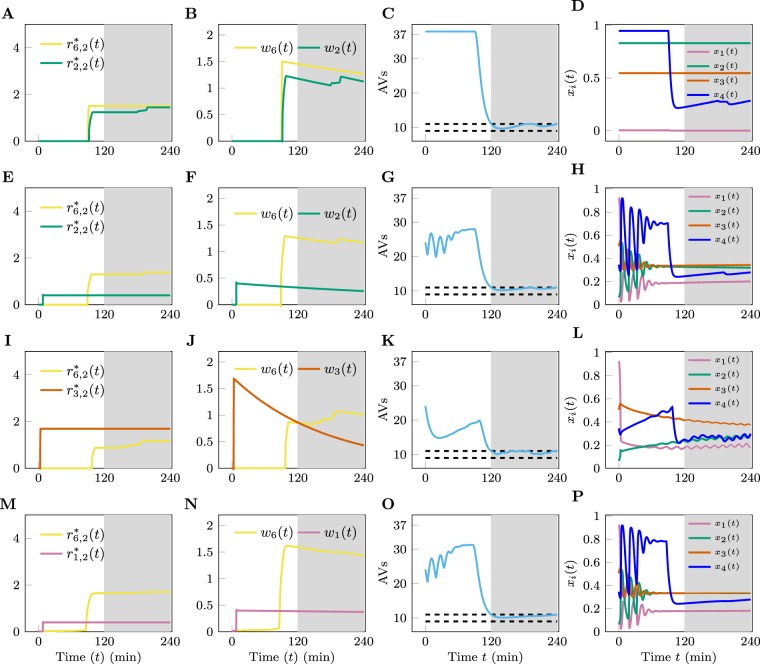
Optimal dual therapies. (**A**–**D**) Panels (A–D) are from a numerical experiment for which we set ${C}_{{\rm{Nu}}}={C}_{{\rm{En}}}=0.1$ and attempt to use a combination of drugs 2 and 6. (**E**–**H**) Panels (E–H) are from a numerical experiment in which we set ${C}_{{\rm{Nu}}}={C}_{{\rm{En}}}=0.6$ and attempt to use a combination of drugs 2 and 6. (**I**–**L**) Panels (I–L) are from a numerical experiment in which we set ${C}_{{\rm{Nu}}}={C}_{{\rm{En}}}=0.6$ and attempt to use a combination of drugs 3 and 6. (**M**–**P**) Panels (M–P) are from a numerical experiment in which we set ${C}_{{\rm{Nu}}}={C}_{{\rm{En}}}=0.6$ and attempt to use a combination of drugs 2 and 6. The plots on the first column are cumulative drug dosages for the dual therapies considered. The plots on the second column are drug concentrations. The plots in the third column show *x*_5_(*t*) and the plots in the fourth, rightmost, column show *x*_1_(*t*), *x*_2_(*t*), *x*_3_(*t*), and *x*_4_(*t*), which we did not attempt to control. In all the simulations, the target value for AV count was 10 (i.e., ${x}_{5}^{f}=10$) and the upper bound on each drug concentration *w*_*i*_ was 2 (i.e., ${w}_{i}^{{\rm{\max }}}=2$). The white region corresponds to the time interval [*t*_0_, *t*_*f*_] when we either upregulate or downregulate the AV count The shaded region corresponds to the time interval [*t*_0_, *t*_*f*_] when the AV count is maintained within the interval ${x}_{5}^{f}\pm \varepsilon$.

Let us now consider dual therapies ($k=2$). The motivation is to identify therapies—protocols involving lower quantities of drugs and faster responses—that are even more efficient than optimal monotherapies. We have evaluated all possible dual therapies (${C}_{2}^{6}=15$) for each of two energy/nutrient stress conditions: ${C}_{{\rm{En}}}={C}_{{\rm{Nu}}}=0.1$ (corresponding to severe stress) and ${C}_{{\rm{En}}}={C}_{{\rm{Nu}}}=0.6$ (corresponding to moderate stress). With an identical control objective and identical constraints ${w}_{i}^{{\rm{\max }}}=2.0$ ${t}_{0}=120$, ${t}_{f}=240$, ${x}_{5}^{f}=10$, and $\varepsilon =1$, we found four pairs of drugs that are each more efficient than the optimal monotherapy with either of the two drugs included in the combination. These dual therapies are illustrated in Fig. 5. Additional results from our analyses of dual therapies are presented in the Supplementary Note and Supplementary Figs S3–S10.

We found that when baseline autophagy is high (${C}_{{\rm{En}}}={C}_{{\rm{Nu}}}=0.1$), the only combination of drugs that can drive AV count down to the target ${x}_{5}^{f}$ is the combination of drugs 2 and 6. The dynamical response of the system is shown in Fig. 5A–D. For this particular combination, either drug alone cannot lower *x*_5_ to 10 without violating one or both of the constraints ${w}_{i} < {w}_{i}^{{\rm{\max }}}$ (*i* = 2, 6). However, with use of drugs 2 and 6 in combination, it is possible to achieve the target AV count because the effects of the drugs are multiplicative (Eq. (1d)) and drug 2 directly affects both MTORC1 (Eq. (1a)) and VPS34 (Eq. (1d)).

Our analysis predicts non-trivial synergistic activities between drugs when the baseline level of autophagy is intermediate (on average) and exhibits oscillatory behavior (${C}_{{\rm{En}}}={C}_{{\rm{Nu}}}=0.6$). The results are summarized in Fig. 5E–P. In this scenario, multiple drug combinations (drugs 1 and 6, 2 and 6, and 3 and 6) are able to downregulate and stabilize *x*_5_, whereas drug 6 alone cannot do so. Using drug 6 alone results in oscillations in *x*_5_, causing a violation of the constraint of Eq. (6c). More interestingly, the optimal application of the drugs reveals a clear sequential protocol: first apply a drug other than drug 6 (1, 2, or 3) to suppress oscillations (see Fig. 5H,L,P), then apply drug 6 to drive AV count down to the desired level. The combination of drugs 1 and 6 is peculiar in that in this case application of drug 1 drives the system out of the oscillatory regime (Fig. 5O) but also upregulates autophagy; subsequent application of drug 6 is effective in downregulating autophagy.

It is important to emphasize that the two drugs acting together in any given combination therapy are, for simplicity, modeled as non-interacting, which may or may not be reasonable, depending on the mechanisms of actions of specific drugs of interest. The drug synergies detected in our analyses arise from the nonlinear dynamics of the regulatory network controlling autophagy. Without the formal framework presented here for therapy design, it would arguably be difficult to identify these synergies.

## Discussion

Here, we have taken up the problem of designing targeted therapies to control a cellular phenotype of cancer cells, namely, their commitment to recycling of cytoplasmic contents through the process of autophagy, as measured by cellular autophagic vesicle (AV) count. Autophagy generates building blocks needed for *de novo* protein synthesis in support of growth (and proliferation). Modulation of autophagy, up or down, in autophagy-addicted cancer cells has the potential to selectively kill these cells^26^.

Our approach was to first construct a mathematical model for autophagy regulation that captures the effects of key physiological stimuli—changes in the supplies of energy and nutrients—and the idealized effects of six available drug types (Eq. (1), Figs. 1–3) and to then pose the question of therapy design as a constrained, optimal control problem (Eqs (4)–(6)). Numerical solution of this problem, through optimization of a control input accounted for in the model (i.e., an adjustable time-dependent drug injection/input rate), yielded monotherapy drug schedules that require a minimum amount of drug, maintain drug concentration below a specified threshold at all times, and that bring about desired effects in the most efficient manner possible (Fig. 4), in a well-defined sense. Furthermore, through the essentially same approach, but with consideration of adjustable time-dependent drug injection/input rates for two different drugs, we were able to predict synergistic drug pairs (Fig. 5).

Optimal monotherapies were found to entail intermittent pulses of drug injection/input at irregular, non-obvious intervals and doses (Fig. 4). These features of optimal drug schedules—the pulsatile nature of drug administration and the irregularity of drug administration in terms of both timing and dosage—appear to be generic and each is discussed in further detail below.

The pulsatile nature of optimal monotherapy arises from the optimal control problem that we posed (Eqs (4)–(6)), which can be viewed as a minimum-fuel problem, in that our control problem calls for usage of a minimal total amount of drug. The rationale for this control objective is that drugs typically have dose-dependent offtarget effects, which may contribute to drug toxicity. Thus, by seeking drug schedules that achieve desired endpoints while using only a minimal total amount of drug, we seek to mitigate the possible negative consequences of offtarget drug effects. Mathematically, our minimum-fuel objective function, Eq. (5), leads to pulsatile drug administration because the Hamiltonian of the optimal control problem is linear in the control inputs *u*_*i*_(*t*), $i\in {{\mathscr{T}}}_{k}$ (see “Pseudo-Spectral Optimal Control” in Supplementary Methods for a detailed derivation). Optimal control problems which have Hamiltonians that are linear in the control input are well-known to have singular arcs, that is, discontinuities jumping between upper and lower bounds of the control input (see Chapter 5 in Kirk^50^ for the derivation of singular arc behavior and the brief overview of this issue in “Pseudo-Spectral Optimal Control” in Supplementary Methods). Because we do not impose an upper bound on *u*_*i*_(*t*), the discontinuities we expect to see are Dirac delta type functions, a pulse of infinite magnitude but infinitesimal width. With the use of numerical methods to find solutions of the optimal control problem, we cannot capture the Dirac delta behavior exactly. Instead, we see finite pulses of finite width, which, while likely suboptimal, are more physically realistic.

Although pulses of drug input are consistent with convenient drug delivery modalities, such as oral administration of a drug in pill form or intravenous injection, optimal schedules do not entail uniform drug doses, nor uniform periods of drug administration. This irregular nature of optimal drug administration depends on the structure of the nonlinear cellular network that controls the synthesis of AVs. In particular, in our model, each drug specifically targets individual nodes of the cellular network, and therefore, different drugs play dynamically distinct roles and cannot be treated as equivalent control inputs. Thus, it may be critically important to better understand the interplay between targeted therapies and archetypical cellular regulatory network dynamics if we are to design the best possible therapies for populations of patients. Because network dynamics can be expected to vary between patients, patient-specific variability in network dynamics, which we have not considered in our analyses here, is a factor that likely affects the efficacy of individualized targeted therapy and that therefore should receive attention in future studies. The study of Fey *et al*.^54^ points to the feasibility of considering patient-specific parameters in mathematical models. In this study, gene expression data available for individual patients were used to set the abundances of gene products in patient-specific models for a cell signaling system. Because mutations can be detected in the tumors of individual patients, effects of oncogenic mutations could also potentially be accounted for in patient-specific models. The study of Rukhlenko *et al*.^6^ provides an example of a study where the effects of an oncogenic mutation were considered in a mathematical model. In the study of Fröhlich *et al*.^55^, gene expression and mutational profiles were both considered in cell line-specific models.

The therapy design approach presented here is flexible and allows for the evaluation of drug combinations. In our analyses, we focused on dual therapies. Somewhat surprisingly, we found several drug pairs that together are more effective than either drug alone (according to our model). These pairs are drug 2 and drug 6 when ${C}_{{\rm{Nu}}}={C}_{{\rm{En}}}=0.1$ (severe energy/nutrient stress) and the combination of drug 6 with drug 1, 2, or 3 when ${C}_{{\rm{Nu}}}={C}_{{\rm{En}}}=0.6$ (moderate energy/nutrient stress). In the latter cases, drug 6 alone is incapable of downregulating autophagy to the desired level, but it sensitizes the network to drugs 1–3 when one of these drugs is used in conjunction with drug 6. According to the model (and its parameterization), the most potent synergistic drug pair is the combination of drugs 2 and 6. With this combination, the total amount of drug 2 used was reduced by more than 5-fold (see the Supplementary Note and Supplementary Fig. S5) in comparison to the case where drug 2 is used optimally in isolation. More striking perhaps is that drug 6 when used alone is incapable of achieving the performance objective. Interestingly, our results provide mechanistic insight into the optimal sequence of drug delivery: therapy is optimal when drug 2 is injected about 80 minutes earlier than drug 6. That is, the best outcome was achieved when first inhibiting MTORC1, thus halting the intrinsic oscillations of the network dynamics, and then only inhibiting VPS34 to reduce synthesis of AVs. It should be noted that in our evaluation of this drug pair, we have assumed that there is no interaction between drugs 2 and 6, an idealization that may not be appropriate for specific examples of drugs of these types.

The same optimal control approach that we have demonstrated for 2-drug combinations can be applied for combinations involving more than two drugs. Indeed, our approach was presented for the general case of *k* drugs used in combination. Our expectation is that effective combinations involving more than two drugs may be more likely to exist than effective combinations involving only two drugs, because controllability would presumably increase with the availability of more drugs. However, finding an effective combination may be more computationally expensive because of the larger number of possible combinations, and 2-drug combinations may be preferable to higher-order combinations because of drug side effects.

As reported by Palmer and Sorger^56^, many clinically used drug combinations are effective for reasons other than drug synergy, which is rare. In essence, the majority of clinically available drug combinations are, for all intents and purposes, equivalent to monotherapy at the level of individual patients. The basis for their effectiveness at the population level is simply that tumors in different subpopulations of patients have distinct drug sensitivities. Thus, new methods for predicting promising, non-obvious synergistic drug combinations, such as the approach reported here, could be helpful in developing combination therapies that derive their effectiveness from drug synergy. Synergistic drug combinations would seemingly offer significant benefits over monotherapy, or what is effectively monotherapy, in terms of delaying or perhaps eliminating the emergence of drug resistance. We note that our analysis identified synergies between pairs of drugs that are predicted to manifest without fine tuning of the doses used or the timing of drug administration. We admit that these predictions could perhaps have been found through an *ad hoc* model analysis. Nevertheless, we see value in leveraging an optimal control framework for model analysis, even if an optimal control strategy is not sought, because with this type of approach it is less likely that interesting behavior will be missed.

There is presently cautious optimism that effective drug combinations will be identified through high-throughput screening experiments^57^, or through learning from data. However, the sheer number of possible drug combinations poses a barrier to experimental discovery of efficacious drug combinations and it is not clear that the data requirements of machine learning approaches can be met in the near term. Thus, it is important to consider alternatives, such as the approach presented here, which leverages available mechanistic understanding of how regulatory protein/lipid kinases influence the synthesis of AVs, which we have consolidated in the form of a mathematical model (Eq. (1)), designed to be useful for computational characterization of drug combinations. We note that our model was formulated specifically for this purpose, and it was not designed to make predictions outside this limited domain of application. Indeed, to facilitate our computational analyses, the model was handcrafted to be as simple as possible while still reproducing key behaviors of more mechanistically detailed models^33,34^. This approach was helpful in making calculations feasible. Unfortunately, to our best knowledge, there are no proven approaches for systematically and automatically deriving a suitable surrogate model for therapy design from a more detailed, mechanistic model of a cellular regulatory network. Pursuit of such a capability seems like an important subject of future research.

Our intent at the start of this study was to investigate how control engineering concepts might be introduced into formal therapy design. Thus, we have only attempted to demonstrate that our methodology is capable of generating interesting (and testable) predictions of effective drug schedules and drug combinations. Development of novel therapies will, of course, require experimental validation of candidate combinations, which is beyond the intended scope of the present study. Thus, we caution that our predictions of optimal drug schedules and synergistic drug combinations are only intended to demonstrate methodology. The merit of this methodology is not in reaching final conclusions but in prioritizing experimental efforts and thereby accelerating experimental validation of targeted therapies. Because kinase inhibitors of each type considered in our analysis are available for experimental characterization and autophagy is a cellular phenotype that can be readily assayed, as in the study of Martin *et al*.^34^ or du Toit *et al*.^58^, a logical next step would be to probe for the predicted drug synergies in cell line experiments. It might be especially interesting to evaluate a combination of an ULK1-specific inhibitor, such as ULK-101^59^, and a VPS34-specific inhibitor, such as VPS34-IN1^60^. We predict that this combination will be synergistic, and the combination targets the two kinases considered in our analysis that are most proximal to the cellular machinery for producing autophagosomes. On the computational side, to increase confidence in predictions, sensitivity analysis techniques tailored for optimal control problems could be applied to characterize the robustness of predictions^61,62^, and experimental design techniques could be applied to aid in generating data useful for reducing parameter uncertainty^63,64^. Several studies strongly support the potential value of formal therapy design^65–67^, and the main contribution here is a new approach to this subject. Two important distinguishing features of this approach are 1) the consideration of a mathematical model for a cellular regulatory network that controls a cellular phenotype and 2) application of sophisticated methods from automatic control theory.

## Methods

### Simulations

Simulations were performed by numerical integration of the model ODEs. The parameter settings used in calculations are provided in the Supplementary Tables S1 and S2.

### Pseudo-Spectral Optimal Control

Optimal control as a field of research combines aspects of dynamical systems, mathematical optimization and the calculus of variations^50^. Together Eqs (5) and (6) form a constrained optimal control problem, which can generally be written as,

8 $$\begin{array}{ll}\mathop{{\rm{\min }}}\limits_{{\bf{u}}(t)} & J({\bf{x}}(t),{\bf{u}}(t),t)={\int }_{{t}_{0}}^{{t}_{f}}\,F\,({\bf{x}}(t),{\bf{u}}(t),t)\,{\rm{d}}t\\ {\rm{s}}.\,{\rm{t}}. & \dot{{\bf{x}}}(t)={\bf{f}}({\bf{x}}(t),{\bf{u}}(t),t)\\  & {{\bf{e}}}^{L}\le {\bf{e}}({\bf{x}}({t}_{0}),{\bf{x}}({t}_{f}),{t}_{0},{t}_{f})\le {{\bf{e}}}^{U}\\  & {{\bf{h}}}^{L}\le {\bf{h}}({\bf{x}}(t),{\bf{u}}(t),t)\le {{\bf{h}}}^{U}\\  & t\in [{t}_{0},{t}_{f}]\end{array}$$

In general, there exists no analytic framework that is able to provide the optimal time traces of the controls and the states in Eq. (8), and so we must resort to numerical techniques.

Pseudo-spectral optimal control (PSOC) has become a popular tool in recent years^68,69^ that has allowed scientists and engineers solve optimal control problems like that of Eq. (8) reliably and efficiently in applications such as guiding autonomous vehicles and maneuvering the international space station^69^. The main concepts of PSOC are summarized here but are explained at length in “Pseudo-Spectral Optimal Control” in Supplementary Methods. See also Supplementary Fig. S11. We define a set of *N* discrete times $\{{\tau }_{i}\}$ $i=0,1,\ldots ,N$ where ${\tau }_{0}=-\,1$ and ${\tau }_{N}=1$ with a mapping between $t\in [{t}_{0},{t}_{f}]$ and $\tau \in [\,-\,1,1]$. The choice of $\{{\tau }_{i}\}$ is key to the convergence of the full discretized problem and so typically they are chosen as the roots of an *N* + 1th order orthogonal polynomial such as Legendre or Chebyshev. In fact, the type of PSOC one uses is typically named after the type of polynomial used to generate the discretization points. Let $\hat{{\bf{x}}}(\tau )={\sum }_{i=0}^{N}\,{\hat{{\bf{x}}}}_{i}{L}_{i}(\tau )$ be an approximation of ${\bf{x}}(\tau )$ where ${L}_{i}(\tau )$ is the *i*th Lagrange interpolating polynomial. The dynamical system is approximated by differentiating the approximation $\hat{{\bf{x}}}(\tau )={\sum }_{i=0}^{N}\,{\hat{{\bf{x}}}}_{i}{L}_{i}(\tau )$ with respect to time.

9 $$\frac{d\hat{{\bf{x}}}}{d\tau }=\sum _{i=0}^{N}\,{{\bf{x}}}_{i}\frac{d{L}_{i}}{d\tau }$$

Let ${D}_{k,i}=\frac{d}{d\tau }{L}_{i}({\tau }_{k})$ so that we may rewrite the original dynamical system constraint in Eq. (8) as the following set of algebraic constraints.

10 $$\sum _{i=0}^{N}\,{D}_{k,i}{{\bf{x}}}_{i}-\frac{{t}_{f}-{t}_{0}}{2}{\bf{f}}({\hat{{\bf{x}}}}_{i},{\hat{{\bf{u}}}}_{i},{\tau }_{i})={\bf{0}},\,k=1,\ldots ,N$$

With the original time-varying states and control inputs now discretized, the dynamical equations approximated with Lagrange interpolating polynomials, and the cost function approximated by a quadrature, the discretized optimal control problem can be expressed as the following nonlinear programming (NLP) problem.

11 $$\begin{array}{ll}\mathop{{\rm{\min }}}\limits_{\begin{array}{c}{{\bf{u}}}_{i}\\ i=0,\ldots ,N\end{array}} & \hat{J}=\frac{{t}_{f}-{t}_{0}}{2}\,\sum _{i=0}^{N}\,{w}_{i}f({\hat{{\bf{x}}}}_{i},{\hat{{\bf{u}}}}_{i},{\tau }_{i})\\ {\rm{s}}.\,{\rm{t}}. & \sum _{i=0}^{N}\,{D}_{k,i}{\hat{{\bf{x}}}}_{i}-\frac{{t}_{f}-{t}_{0}}{2}{\bf{f}}({\hat{{\bf{x}}}}_{k},{\hat{{\bf{u}}}}_{k},{\tau }_{k})={\bf{0}},\,k=0,\ldots ,N\\  & {{\bf{e}}}^{L}\le {\bf{e}}({\hat{{\bf{x}}}}_{0},{\hat{{\bf{x}}}}_{N},{\tau }_{0},{\tau }_{N})\le {{\bf{e}}}^{U}\\  & {{\bf{h}}}^{L}\le {\bf{h}}({\hat{{\bf{x}}}}_{k},{\hat{{\bf{u}}}}_{k},{\tau }_{k})\le {{\bf{h}}}^{U},\,k=0,\ldots ,N\\  & {t}_{i}=\frac{{t}_{f}-{t}_{0}}{2}{\tau }_{i}+\frac{{t}_{f}+{t}_{0}}{2}\end{array}$$

We used ${\mathscr{P}}{\mathscr{S}}{\mathscr{O}}{\mathscr{P}}{\mathscr{T}}$^35^, an open-source PSOC toolbox written in C++, to perform the PSOC discretization procedure.

The NLP problem of Eq. (11) can be solved with a number of different techniques, but here we use an interior point algorithm^70^ as implemented in the open-source C++ software Ipopt^71^.

## Supplementary information

Supplementary Information

Supplementary Dataset

## Data Availability

Problem-specific software used in this study is provided as Supplementary Data.
