# Supplementary material for: Prediction of Optimal Drug Schedules for Controlling Autophagy
Source: Sci Rep. 2019 Feb 5;9:1428. doi: 10.1038/s41598-019-38763-9 (PMC6363771; doi:10.1038/s41598-019-38763-9)
Supplement: Supplementary file 1 — Supplementary Information [file 41598_2019_38763_MOESM1_ESM.pdf]

Supplementary Information—Prediction of Optimal Drug Schedules for Controlling Autophagy

Afroza Shirin[1,+], Isaac S. Klickstein[1,+], Song Feng[2,+], Yen Ting Lin[2,+], William S. Hlavacek[2,+,*], and Francesco Sorrentino[1,+,*]

[1]Mechanical Engineering Department, University of New Mexico, Albuquerque, NM 87131
[2]Theoretical Biology and Biophysics Group, Theoretical Division and Center for Nonlinear Studies, Los Alamos National Laboratory, Los Alamos, NM 87545
[+]These authors contributed equally.
[*]Corresponding authors: wish@lanl.gov (W.S.H.), fsorrent@unm.edu (F.S.)

Supplementary Methods

Formulation of the Model

Formulation of Eq. (1) was guided by the models of Szymańska et al.[1] (Ref. 33 in the main text) and Martin et al.[2] (Ref. 34 in the main text) mainly as follows. The model of Eq. (1) was formulated and parameterized so as to allow the model to predict oscillatory induction of autophagy in response to intermediate drug, energy, and nutrient stress inputs (as illustrated in Figs. 2 and 3), in accord with the predictions of the model of Szymańska et al.[1]. Moreover, as in both models considered by Martin et al.[2], Eq. (1) takes AVs to be turned over constitutively via a pseudo first-order degredative process. Another factor that drove model formulation and parameterization was the availability of measured AV dynamics induced by MTORC1 inhibition[2]. Eq. (1) was parameterized so as to reproduce the essential aspects of these dynamics (see below for more discussion).

Equation (1) differs from the earlier models of Szymańska et al.[1] and Martin et al.[2] mainly as follows. In the model of Szymańska et al.[1], the regulatory influences depicted in Fig. 1 (e.g., mutual inhibition of MTORC1 and ULK1 and negative feedback from ULK1 to AMPK) are not explicitly represented, as is the case in the model of Eq. (1), where regulatory influences on enzymatic activities are represented explicitly using Hill functions. Rather, in the model of Szymańska et al.[1], regulatory influences emerge from formal representations of the biomolecular interactions considered in the model, which are termed rules[3]. In other words, Eq. (1) provides a model of regulatory influences and their effects, whereas the model of Szymańska et al.[1] provides a model of biomolecular interactions and their effects, which include emergent regulatory influences. The rules of the model of Szymańska et al.[1] can be processed automatically by the BioNetGen software package[4] to obtain a system of 173 coupled ordinary differential equations (ODEs). These equations account for various complexes (e.g., a complex of AMPK and ULK1 that is generated when AMPK docks to a particular site in ULK1) and protein phosphoforms. In contrast, the model of Eq. (1) does not track these details. Rather, it simply tracks the activities of AMPK, MTORC1, and ULK1 (and also the activity of VPS34, which was not considered by Szymańska et al.[1]). In the model of Szymańska et al.[1], AMPK, MTORC1, and ULK1 each has numerous states. In contrast, in the model of Eq. (1), these protein states are reduced to just two for each protein: active or inactive.

Although the model of Szymańska et al.[1] provides a mechanistically detailed representation of biomolecular interactions, it does not include a representation of autophagic vesicle (AV) population dynamics. To include a representation of AV population dynamics in Eq. (1), we started with the simple representation of AV production and clearance used in the AV population dynamics model of Martin et al.[2]:

$$\frac{dV}{dt} = P^* - cV,$$

where V is cellular AV count, P^* is a condition-dependent zero-order rate constant for AV production, and c is a pseudo first-order rate constant for clearance of AVs. In our model, we modified this equation by allowing the production rate to be time dependent. In Eq. (1) the rate of AV production is a linear function of VPS34 activity, $x_4(t)$. In other words, the rate of AV production is given by $k_3x_4(t)$ (vs. a constant, P^*).

Parameter settings are summarized in Supplementary Tables S1 and S2. These settings are not uniquely determined by data; they were guided by the considerations explained below.

Parameter settings for parameters in the h and H Hill functions were determined first, as follows. For each Hill function, we initially set $r_b = 0$, $r_m = 1$, $\theta = 0.5$, and $n = 2$. (We omit indices in referring to these parameters for convenience.) We then varied parameter values (by hand tuning) to obtain qualitative behavior consistent with that predicted by the model of Szymańska et al.[1]. The behaviors of the two models are compared directly in Supplementary Fig. S1. In panels *A* and *B* of Supplementary Fig. S1, AV count (x_5) and ULK1 activity (x_2) are shown, respectively, as a function of time. Initially, in these plots, we consider a nutrient/energy replete condition ($C_{\text{En}} = C_{\text{Nu}} = 1$) without rapamycin (or any other drug). A low dose of rapamycin is added at time $t = 100$ min and then a high dose of rapamycin is added at time $t = 200$ min. As can be seen, x_5 (Supplementary Fig. S1*A*) and x_2 (Supplementary Fig. S1*B*) initially have steady low values. After the initial introduction of rapamycin, these quantities begin to oscillate. After the second addition of rapamycin, the two quantities have steady high values. This behavior is qualitatively the same as the behavior predicted by the model of Szymańska et al.[1] (Supplementary Fig. S1*C*). It should be noted that the study of Szymańska et al.[1] did not establish that the AMPK-MTORC1-ULK1 network actually exhibits oscillatory behavior; this study only showed that oscillatory behavior is a possible consequence of known regulatory mechanisms. By requiring Eq. (1) to reproduce the qualitative nonlinear dynamics of the model of Szymańska et al.[1], we made the optimal control problem considered here more of a challenging test of our methodology.

Next, parameter settings for the rate constants k_1, k_2, k_3 and k_4 were determined (again through hand tuning). In the study of Martin et al.[2], AV population dynamics were monitored after cells in a nutrient/energy replete condition were treated with a dose of rapamycin or AZD8055 (a catalytic MTOR inhibitor) sufficient to fully inhibit MTORC1 activity. We selected values for the rate constants that allow the model of Eq. (1) to roughly reproduce the observed dynamics induced by MTORC1

inhibition in the study of Martin et al.[2]. The behaviors predicted by Eq. (1) and the model of Martin et al.[2] are directly compared in panels *D* and *E* of Supplementary Fig. S1. The AV population dynamics model of Martin et al.[2] can be written as follows: $dV/dt = (1 + k\delta)P - cV$, where $\delta = 0$ indicates a 0 dose of MTORC1 inhibitor, $\delta = 1$ indicates a saturating dose of MTORC1 inhibitor, P is the baseline rate of AV production, and $(1 + k)P$ is the induced rate of AV production stimulated by a saturating dose of MTORC1 inhibitor. By varying δ from 0 to 1, we obtain the plots shown in Supplementary Fig. S1*E*. Note that AV dynamics at intermediate values for δ are not oscillatory, as we would expect from the analysis of Szymańska et al.[1]. In contrast, Eq. (1) does predict oscillatory AV dynamics at intermediate doses of MTORC1 inhibitor (Supplementary Fig. S1*D*). Importantly, as desired, Eq. (1) makes predictions that are in qualitative agreement with the model of Martin et al.[2], in that both models predict that AV dynamics stimulated by MTORC1 inhibitor treatment unfold on a similar timescale and that the maximal range of regulation is similar. In Supplementary Fig. S2, we directly compare the AV dynamics predicted by Eq. (1) with AV dynamics measured by Martin et al.[2]. As can be seen, Eq. (1) is roughly consistent with the data.

Finally, parameter settings for the drug clearance rate constants in Eq. (1) ($\delta_1, \ldots, \delta_6$) were set in accordance with measured drug lifetimes reported in the literature, which have half-lives ranging from approximately 1 to 40 h. See Supplementary Table S2 and references cited therein. With this approach, the different drugs considered have different pharmacokinetics, arguably making the optimal control problem more realistic.

Pseudo-Spectral Optimal Control

We present here a brief overview of the theory of pseudo-spectral optimal control (PSOC). Before discussing the PSOC framework, we briefly review optimal control as well as the difficulties that arise when attempting to solve a general optimal control problem (OCP) analytically. Afterwards, we describe how PSOC discretizes the OCP, approximating the original OCP as a nonlinear programming (NLP) problem. Approximating the original problem as an NLP is beneficial because there exists a vast literature and many pieces of software capable of solving large-scale NLPs efficiently. Finally, we discuss our choices of software, all of which are open-source, and briefly discuss the algorithms they implement.

Optimal Control

The field of optimal control combines aspects of dynamical systems, optimization, and calculus of variations[5]. In words, an optimal control problem is solved by finding a time varying control input $\mathbf{u}(t)$ that minimizes a quantity $J(\mathbf{x}, \mathbf{u}, t)$ subject to a system's dynamics and other constraints.

General Problem

Define the states of the system as $\mathbf{x}(t) \in \mathbb{R}^n$, the control inputs as $\mathbf{u}(t) \in \mathbb{R}^m$, and time $t \in [t_0, t_f]$ where $t_0 < t_f$. The typical form of an optimal control problem for a continuous-time system can be written as,

$$\begin{aligned} \min_{\mathbf{u}(t)} \quad & J(\mathbf{x}(t), \mathbf{u}(t), t) = E\left(\mathbf{x}(t_0), \mathbf{x}(t_f), t_0, t_f\right) + \int_{t_0}^{t_f} F\left(\mathbf{x}(t), \mathbf{u}(t), t\right) dt \\ \text{s.t.} \quad & \dot{\mathbf{x}}(t) = \mathbf{f}(\mathbf{x}(t), \mathbf{u}(t), t) \\ & \mathbf{e}^L \le \mathbf{e}(\mathbf{x}(t_0), \mathbf{x}(t_f), t_0, t_f) \le \mathbf{e}^U \\ & \mathbf{h}^L \le \mathbf{h}(\mathbf{x}(t), \mathbf{u}(t), t) \le \mathbf{h}^U \\ & t \in [t_0, t_f] \end{aligned} \tag{S1}$$

The objective function (or cost function) $J(\mathbf{x}, \mathbf{u}, t)$ is composed of two parts, (i) $E : \mathbb{R}^n \times \mathbb{R}^n \times \mathbb{R} \times \mathbb{R} \mapsto \mathbb{R}$ which is a cost associated with the endpoint behavior of the system $\mathbf{x}(t_0)$ and $\mathbf{x}(t_f)$, and (ii) $F : \mathbb{R}^n \times \mathbb{R}^m \times \mathbb{R} \mapsto \mathbb{R}$ which is a running cost over the entire time interval $[t_0, t_f]$. The system dynamics is described by the function $\mathbf{f} : \mathbb{R}^n \times \mathbb{R}^m \times \mathbb{R} \mapsto \mathbb{R}^n$. Constraints on the endpoints ($\mathbf{x}(t_0)$ and/or $\mathbf{x}(t_f)$) are described by $\mathbf{e} : \mathbb{R}^n \times \mathbb{R}^n \times \mathbb{R} \times \mathbb{R} \mapsto \mathbb{R}^e$. While we only specify initial conditions, more complicated relations between the endpoints of the states can be specified as well. Finally, path constraints, such as bounds on the states or control inputs, are described by $\mathbf{h} : \mathbb{R}^n \times \mathbb{R}^n \times \mathbb{R} \mapsto \mathbb{R}^h$.

Notation for Therapies

Let $\mathcal{D} = \{1, 2, 3, 4, 5, 6\}$ denote the possible drugs we may use (described in the main text) and $\mathcal{T}_k \subseteq \mathcal{D}$ denote the drugs chosen for our therapy such that $|\mathcal{T}_k| = k$. Let $\mathbf{w}(t) \in \mathbb{R}^k$ denote the drug concentrations and $\mathbf{u}(t) \in \mathbb{R}^k$ denote the drug injection rates for *only those drugs chosen to be in the therapy*. For example, if we consider the dual therapy $\mathcal{T}_2 = \{3, 6\}$, then

$$\mathbf{w}(t) = \begin{bmatrix} w_3(t) \\ w_6(t) \end{bmatrix}, \quad \mathbf{u}(t) = \begin{bmatrix} u_3(t) \\ u_6(t) \end{bmatrix} \tag{S2}$$

Those drugs not chosen to be in $\mathcal{T}_k$ are denoted $\mathcal{D}\backslash\mathcal{T}_k$. In the example where $\mathcal{T}_k = \{3,6\}$, those drugs not used are $\mathcal{D}\backslash\mathcal{T}_k = \{1,2,4,5\}$. If a drug $i \in \mathcal{D}\backslash\mathcal{T}$ then we set $w_i(t) = 0$ for all time t.

The drug concentrations appear in the dynamical equations as inhibitory Hill functions $H(w_i(t))$.

$$H(w_i(t)) = r_{m,i} - (r_{m,i} - r_{b,i})\frac{w_i^{n_i}(t)}{w_i^{n_i}(t) + \theta^{n_i}} \tag{S3}$$

Note that if $i \notin \mathcal{T}_k$, then, as stated previously, $w_i(t) = 0$, and so, by Eq. (S3), $H(w_i(t)) = 1$ for all time t.

The Minimum Drug OCP

In the main text, we present a *multi-phase optimal control problem*, i.e., two optimal control problems linked together by enforcing continuity at their interface. Despite this added complexity, we can develop a set of necessary conditions for each phase individually and so for now we focus on the single phase problem. We will return to the multi-phase problem in the next section that covers the discretization procedure.

Either phase of the OCP presented in the main text can be mapped to the general formulation presented in Eq. (S1) with the following definitions.

- The state variables $\mathbf{x}(t) = \begin{bmatrix} x_1(t) & x_2(t) & x_3(t) & x_4(t) & x_5(t) & \mathbf{w}^T(t) \end{bmatrix}^T \in \mathbb{R}^{5+k}$ and the control input $\mathbf{u}(t) \in \mathbb{R}^k$ so that $n = 5 + k$ and $m = k$.
- The cost function $J = \int_{t_0}^{t_f} u_i(t)dt$ (see Eq. (1) in the main text) so that, from Eq. (S1), $E \equiv 0$ and $F = \sum_{i\in\mathcal{T}} u_i(t)$.
- The system dynamics, as presented in Eq. (1), are rewritten here,

$$\begin{aligned}\dot{\mathbf{x}}(t) = \begin{bmatrix} \dot{x}_1(t) \\ \dot{x}_2(t) \\ \dot{x}_3(t) \\ \dot{x}_4(t) \\ \dot{x}_5(t) \\ \dot{\mathbf{w}}(t) \end{bmatrix} &= \mathbf{f}(\mathbf{x}(t),\mathbf{u}(t)) = \bar{\mathbf{f}}(\mathbf{x}(t)) + B\mathbf{u}(t) \\ &= \begin{bmatrix} (1-x_1)C_{\text{Nu}}H(w_1)H(w_2) - x_1h_{12}(x_2)h_{13}(x_3) \\ (1-x_2)h_{23}(x_3)H(w_3) - x_2h_{21}(x_1) \\ (1-x_3)k_1H(w_4) - C_{\text{En}}x_2x_3H(w_5) \\ (1-x_4)h_{42}(x_2)H(w_2)H(w_6) - k_2x_4 \\ k_3x_4 - k_4x_5 \\ -\Delta\mathbf{w}(t) \end{bmatrix} + \begin{bmatrix} \mathbf{0}_k^T \\ \mathbf{0}_k^T \\ \mathbf{0}_k^T \\ \mathbf{0}_k^T \\ \mathbf{0}_k^T \\ I_k \end{bmatrix}\mathbf{u}(t)\end{aligned} \tag{S4}$$

  where $\mathbf{0}_k$ is a vector of all zeros of length k, I_k is the identity matrix of order k, and Δ is a diagonal matrix with the corresponding rates δ_i on the diagonal if $i \in \mathcal{T}$. For example, if $\mathcal{T} = \{3,6\}$, then

$$\Delta = \begin{bmatrix} \delta_3 & 0 \\ 0 & \delta_6 \end{bmatrix} \tag{S5}$$

  Also, note that if $i \notin \mathcal{T}$, then $w_i(t) \equiv 0$ and $H(w_i(t)) = 1$.

- The only endpoint constraints are set at the initial time,

$$\mathbf{e}(\mathbf{x}(t_0),\mathbf{x}(t_f),t_0,t_f) = \begin{bmatrix} x_1(0) \\ x_2(0) \\ x_3(0) \\ x_4(0) \\ x_5(0) \\ \mathbf{w}(0) \end{bmatrix}, \quad \mathbf{e}^L = \mathbf{e}^U = \begin{bmatrix} x_{1,0} \\ x_{2,0} \\ x_{3,0} \\ x_{4,0} \\ x_{5,0} \\ \mathbf{0}_k \end{bmatrix} \tag{S6}$$

  where $x_{i,0}$ is chosen to either be the steady state value of the system in the absence of control inputs or the time-average of the time evolution of the system if the dynamics, in the absence of control inputs, is oscillatory. We assume there is no drug present initially so $w_i(0) = 0$, $i \in \mathcal{D}$.

- Finally, the path constraints consist of upper bounds on the drug concentrations and possibly a lower and/or upper bound on the AVs.

$$\mathbf{h}(\mathbf{x}(t),\mathbf{u}(t),t) = \begin{bmatrix} x_5(t) \\ \mathbf{w}(t) \\ \mathbf{u}(t) \end{bmatrix}, \quad \mathbf{h}^L = \begin{bmatrix} x_5^L \\ \mathbf{0}_k \\ \mathbf{0}_k \end{bmatrix}, \quad \mathbf{h}^U = \begin{bmatrix} x_5^U \\ w^{\max}\mathbf{1}_k \\ \infty \end{bmatrix} \tag{S7}$$

where, for the first phase, $x_5^L = 0$ and $x_5^U = \infty$ but for the second phase we choose $x_5^L = x_5^f - \epsilon$ and $x_5^U + \epsilon$. Also, the upper bound on the drug concentration is chosen to be identical for all drugs in the therapy.

Solving Eq. (S1) is not a trivial task, and typically there exists no closed form solution. Instead one typically must turn to numerical methods, such as PSOC, which we will discuss in the subsequent subsections in some detail. Nonetheless, one can derive a set of necessary conditions that any solution to Eq. (S1) must satisfy using Pontryagin's minimum principle[5]. Developing these types of necessary conditions allows us to construct a set of validation criteria with which we may test the quality of any solution returned by our numerical methods.

A full derivation of Pontryagin's minimum principle is beyond the scope of this work but it is readily available in many standard texts[5]. Here, we present the main results surrounding the Hamiltonian constructed from Eq. (S1).

Minimizing the Hamiltonian

Define a vector of time-varying costates (or adjoint variables) as $\boldsymbol{\lambda}(t) = \begin{bmatrix} \boldsymbol{\lambda}_\mathbf{x}^T(t) & \boldsymbol{\lambda}_\mathbf{w}^T(t) \end{bmatrix}^T \in \mathbb{R}^{5+k}$ so that $\boldsymbol{\lambda}_\mathbf{x}(t) \in \mathbb{R}^5$ and $\boldsymbol{\lambda}_\mathbf{w}(t) \in \mathbb{R}^k$. The Hamiltonian of the OCP in Eq. (S1) is defined as,

$$\begin{aligned} H(\boldsymbol{\lambda},\mathbf{x},\mathbf{u},t) &= F(\mathbf{x},\mathbf{u},t) + \boldsymbol{\lambda}^T\mathbf{f}(\mathbf{x},\mathbf{u},t) \\ &= \sum_{i\in\mathcal{T}} u_i + \boldsymbol{\lambda}^T\bar{\mathbf{f}}(\mathbf{x}) + \boldsymbol{\lambda}B\mathbf{u} \end{aligned} \tag{S8}$$

where $\boldsymbol{\lambda}(t) \in \mathbb{R}^n$ are the costates (or adjoint variables). A solution to Eq. (S1) must also be a solution of the following minimization problem.

$$\begin{aligned} \min_{\mathbf{u}(t)} \quad & H(\boldsymbol{\lambda},\mathbf{x},\mathbf{u},t) \\ \text{s.t.} \quad & \mathbf{h}^L \le \mathbf{h}(\mathbf{x},\mathbf{u},t) \le \mathbf{h}^U \end{aligned} \tag{S9}$$

To solve Eq. (S9), we define the associated Lagrangian,

$$\begin{aligned} \bar{H}(\boldsymbol{\mu},\boldsymbol{\lambda},\mathbf{x},\mathbf{u},t) &= H(\boldsymbol{\lambda},\mathbf{x},\mathbf{u},t) + \boldsymbol{\mu}^T\mathbf{h}(\mathbf{x},\mathbf{u},t) \\ &= \sum_{i\in\mathcal{T}} u_i + \boldsymbol{\lambda}^T\bar{\mathbf{f}}(\mathbf{x}) + \boldsymbol{\lambda}^T B\mathbf{u} + \mu_{x_5}x_5 + \boldsymbol{\mu}_\mathbf{w}^T\mathbf{w} + \boldsymbol{\mu}_\mathbf{u}^T\mathbf{u} \end{aligned} \tag{S10}$$

where $\boldsymbol{\mu} = \begin{bmatrix} \mu_{x_5} & \boldsymbol{\mu}_\mathbf{w}^T & \boldsymbol{\mu}_\mathbf{u}^T \end{bmatrix}^T \in \mathbb{R}^h$ is the copath vector with components associated with the components of the vector of path constraints in (S7). A solution to Eq. (S9), and thus to our original OCP, must satisfy,

$$\frac{\partial \bar{H}}{\partial \mathbf{u}} = \mathbf{1}_k + B^T\boldsymbol{\lambda} + \boldsymbol{\mu}_\mathbf{u} = \mathbf{0} \tag{S11}$$

where the costates evolve according to the dynamical equation,

$$\dot{\boldsymbol{\lambda}} = -\frac{\partial \bar{H}}{\partial \mathbf{x}} = -\left(\frac{\partial \bar{\mathbf{f}}}{\partial \mathbf{x}}\right)^T \boldsymbol{\lambda} + \begin{bmatrix} \mathbf{0}_4 \\ \mu_{x_5} \\ \boldsymbol{\mu}_\mathbf{w} \end{bmatrix} \tag{S12}$$

The optimal control input $u_i(t)$, $i \in \mathcal{T}$, must satisfy the complementarity condition[6,7]

$$\begin{cases} u_i(t) = 0 & \text{if} \quad \mu_i(t) < 0 \\ u_i(t) \ge 0 & \text{if} \quad \mu_i(t) = 0 \\ u_i(t) \to \infty & \text{if} \quad \mu_i(t) > 0 \end{cases} \tag{S13}$$

Combining Eqs. (S11) and (S13), we can relate $\boldsymbol{\mu}_{\mathbf{u}}$ to the time-varying costates by noting from the structure of B, $B^T\boldsymbol{\lambda} = \boldsymbol{\lambda}_{\mathbf{w}}$ so that,

$$\boldsymbol{\mu}_{\mathbf{u}}(t) = -\mathbf{1}_k - \boldsymbol{\lambda}_{\mathbf{w}}(t) \tag{S14}$$

Thus, if $\lambda_{w_i} > -1$ then $u_i = 0$, but if $\lambda_{w_i} = -1$, then all we can say is that $u_i \geq 0$. When $\lambda_{w_i} > -1$, the optimal control is said to have a *singular arc* (see chapter 5 in[5]). Despite the technical difficulties, we have arrived at our first set of validation conditions, that is,

$$u_i \cdot (\lambda_{w_i} - 1) = 0, \quad \forall i \in \mathcal{T} \tag{S15}$$

Let us now assume that we have solved Eq. (S9), that is,

$$\mathcal{H}(t) = \min_{\mathbf{u}\in\mathbb{U}} H(\boldsymbol{\lambda}, \mathbf{x}, \mathbf{u}, t) \tag{S16}$$

where $\mathbb{U}$ is the set of feasible control inputs, i.e., they satisfy all of the constraints imposed by Eq. (S1). The evolution of the Hamiltonian at the optimal solution can be written,

$$\frac{d\mathcal{H}}{dt} = \frac{\partial H}{\partial t} \tag{S17}$$

where, since in our OCP, H does not explicitly depend on time, we expect that $d\mathcal{H}/dt = 0$ and so $\mathcal{H}$ should be constant. This is the second validation condition.

While in the paper and the supplementary sections we display time traces of the states and the control inputs as they are the quantities of interest to the general reader, we are also able to access the costate and copath time traces, as well as the time trace of the Hamiltonian. In Fig. S11 we show a typical set of output that we use for measuring the quality of our returned numerical solution. The sample shows a monotherapy where $\mathcal{T} = \{4\}$. Panel (a) shows the level of AVs, $x_5(t)$, and panel (b) shows the drug concentration $w_4(t)$. Panel (c) contains the copath associated with the level of AVs, $\mu_{x_5}(t)$. Note that during the first phase when there is no finite bound on $x_5(t)$ the copath $\mu_{x_5}(t) = 0$, while during second phase if $\mu_{x_5}(t) \neq 0$ then $x_5(t) = x_5^f \pm \epsilon$. In panel (d) we plot the other copath $\mu_{w_4}(t)$. The control input $u_4(t)$ itself is shown in panel (e) along with the costate $\lambda_{w_4}(t)$ in panel (f). Note that the times at which $u_4(t) > 0$ correspond to times when $\lambda_{w_4}(t) = -1$ as expected. Panel (g) plots the time evolution of the Hamiltonian evaluated at the optimal solution. Note that the y-axis is scaled by 10^{-2}. We see that $\mathcal{H} \approx const$ within each phase, with a jump occurring at the interface between the two phases. As we cannot say anything about the value of the Hamiltonian at the interface, a discontinuity at this point in time can be expected.

Discretization of the OCP

As presented in the previous subsection, we have seen that the set of necessary conditions which must be satisfied consist of a system of coupled nonlinear differential equations for $\mathbf{x}(t)$ and $\boldsymbol{\lambda}(t)$ along with a set of non-trivial constraints. Searching for an analytic solution is unlikely to be successful and so instead we turn to pseudospectral optimal control (PSOC).

In short, PSOC is a methodology by which one may discretize an OCP, approximating the integrals by quadratures and the time-varying states and control inputs with interpolating polynomials.

The key to PSOC is choosing the discretization points properly. Let $\{\tau_i\}$, $i = 0, \ldots, N$, denote the discretization points. Typically these are chosen as the roots of an orthogonal polynomial such as a Legendre polynomial or a Chebyshev polynomial of order N. For some popular choices of discretization schemes see[8]. For concreteness, we will assume that $\tau_0 = -1$ and $\tau_N = 1$, i.e., we are using a discretization scheme that includes the endpoints and is normalized by the mapping,

$$t = \frac{t_f - t_0}{2}\tau + \frac{t_f + t_0}{2} \tag{S18}$$

For the discretization scheme chosen, we also compute the associated quadrature weights. For instance, if we choose the roots of a Legendre polynomial as the discretization scheme, the associated quadrature weights can be found in the typical way for Gauss quadrature. The time-varying states and control inputs are found by approximating them with a Lagrange interpolating polynomial.

$$\begin{aligned} \mathbf{x}(\tau) &\approx \hat{\mathbf{x}}(\tau) = \sum_{i=0}^{N} \hat{\mathbf{x}}_i L_i(\tau) \\ \mathbf{u}(\tau) &\approx \hat{\mathbf{u}}(\tau) = \sum_{i=0}^{N} \hat{\mathbf{u}}_i L_i(\tau) \end{aligned} \tag{S19}$$

The Lagrange interpolating polynomials are defined as,

$$L_i(\tau) = \prod_{j=0, j\neq i}^{N} \frac{\tau - \tau_j}{\tau_i - \tau_j} \tag{S20}$$

Note that the Lagrange interpolating polynomials satisfy the isolation property, that is, $L_i(\tau_j) = \delta_{i,j}$. We can thus construct a set of algebraic equations corresponding to the discretization points $\{\tau_i\}$. Define $D_{k,i} = \frac{dL_i}{d\tau}(\tau_k)$ so that the derivative of the states at the discretization points can be approximated as,

$$\dot{\hat{\mathbf{x}}}(\tau_k) = \sum_{i=0}^{N} \hat{\mathbf{x}}_i D_{k,i} \tag{S21}$$

With Eqs. (S19) and (S21), we can approximate the original system of n differential equations as $n(N+1)$ algebraic equations.

$$\begin{aligned} \sum_{i=0}^{N} D_{k,i}\hat{\mathbf{x}}_i - \frac{t_f - t_0}{2}\mathbf{f}(\hat{\mathbf{x}}_k, \hat{\mathbf{u}}_k, \tau_k) = \mathbf{0}_n, \quad k = 1, \dots, N \\ \hat{\mathbf{x}}_N - \hat{\mathbf{x}}_0 - \sum_{k=1}^{N}\sum_{i=0}^{N} w_k D_{k,i}\hat{\mathbf{x}}_i = \mathbf{0}_n \end{aligned} \tag{S22}$$

The last set of algebraic constraints arise from the consistency condition $\int_{t_0}^{t_f} \dot{\mathbf{x}}(t)dt = \mathbf{x}(t_f) - \mathbf{x}(t_0)$. Similarly to the consistency condition, the integral in the cost function is approximated as,

$$J = \int_{t_0}^{t_f} F(\mathbf{x}, \mathbf{u}, t) \approx \hat{J} = \frac{t_f - t_0}{2} \sum_{k=1}^{N} F(\hat{\mathbf{x}}_k, \hat{\mathbf{u}}_k, \tau_k) \tag{S23}$$

The discretized approximation of the original OCP is compiled into the following nonlinear programming (NLP) problem.

$$\begin{aligned} \min_{\mathbf{u}_i} \quad & \hat{J} = \frac{t_f - t_0}{2} \sum_{k=1}^{N} F(\hat{\mathbf{x}}_k, \hat{\mathbf{u}}_k, \tau_k) \\ \text{s.t.} \quad & \sum_{i=0}^{N} D_{k,i}\hat{\mathbf{x}}_i - \frac{t_f - t_0}{2}\mathbf{f}(\hat{\mathbf{x}}_k, \hat{\mathbf{u}}_k, \tau_k) = \mathbf{0},\ k = 1, \dots, N \\ & \hat{\mathbf{x}}_N - \hat{\mathbf{x}}_0 - \sum_{k=1}^{N}\sum_{i=0}^{N} w_k D_{k,i}\hat{\mathbf{x}}_i = \mathbf{0} \\ & \mathbf{e}^L \leq \mathbf{e}(\hat{\mathbf{x}}_0, \hat{\mathbf{x}}_N, \tau_0, \tau_N) \leq \mathbf{e}^U \\ & \mathbf{h}^L \leq \mathbf{h}(\hat{\mathbf{x}}_k, \hat{\mathbf{u}}_k, \tau_k) \leq \mathbf{h}^U \end{aligned} \tag{S24}$$

With the above results, we now present the application to the full multi-phase optimal control problem. In general, let us assume there are p phases where $p = 2$ in our problem. Each phase is active within the interval $t \in [t_0^{(p)}, t_f^{(p)}]$. In each phase there is a cost function $J^{(p)}$, a dynamical system $\mathbf{f}^{(p)}$, a set of endpoint constraints $\mathbf{e}^{(p)}$, and a set of path constraints $\mathbf{h}^{(p)}$. If two phases, p and q, are linked, then there also exists a set of linkage constraints $\Phi^{(p,q)}$.

$$\begin{aligned} \min_{\mathbf{u}^{(p)}} \quad & \sum_{p=1}^{P} J^{(p)} = \sum_{p=1}^{P} \int_{t_0^{(p)}}^{t_f^{(p)}} F^{(p)}(\mathbf{x}^{(p)}, \mathbf{u}^{(p)}, t)dt \\ \text{s.t.} \quad & \dot{\mathbf{x}}^{(p)}(t) = \mathbf{f}^{(p)}(\mathbf{x}^{(p)}, \mathbf{u}^{(p)}, t) \\ & \mathbf{h}^{L,(p)} \leq \mathbf{h}^{(p)}(\mathbf{x}^{(p)}, \mathbf{u}^{(p)}, t) \leq \mathbf{h}^{U,(p)} \\ & \mathbf{e}^{L,(p)} \leq \mathbf{e}^{(p)}(\mathbf{x}^{(p)}(t_0^{(p)}), \mathbf{x}^{(p)}(t_f^{(p)}), t_0^{(p)}, t_f^{(p)}) \leq \mathbf{e}^{U,(p)} \\ & \Phi^{L,(p,q)} \leq \Phi^{(p,q)}(\mathbf{x}^{(p)}, \mathbf{x}^{(q)}, \mathbf{u}^{(p)}, \mathbf{u}^{(q)}) \leq \Phi^{U,(p,q)} \end{aligned} \tag{S25}$$

Each phase is discretized with its own set of points, $\{\tau_i^{(p)}\}$ so that,

$$\mathbf{x}^{(p)}(\tau) \approx \hat{\mathbf{x}}^{(p)}(\tau) = \sum_{i=1}^{N} \hat{\mathbf{x}}_i^{(p)} L_i(\tau) \tag{S26}$$

so that the full multi-phase NLP is,

$$\begin{aligned}
\min_{\mathbf{u}_i^{(p)}} \quad & \sum_{p=1}^{P} \frac{t_f^{(p)} - t_0^{(p)}}{2} \sum_{k=1}^{N} F^{(p)}(\hat{\mathbf{x}}_k^{(p)}, \hat{\mathbf{u}}_k^{(p)}, \tau_k) \\
\text{s.t.} \quad & \sum_{i=0}^{N} D_{k,i} \hat{\mathbf{x}}_i^{(p)} - \frac{t_f^{(p)} - t_0^{(p)}}{2} \mathbf{f}^{(p)}(\hat{\mathbf{x}}_k^{(p)}, \hat{\mathbf{u}}_k^{(p)}, \tau_k) = \mathbf{0}_n, \quad p = 1, \dots, P, \quad k = 1, \dots, N \\
& \hat{\mathbf{x}}_N^{(p)} - \hat{\mathbf{x}}_0^{(p)} - \frac{t_f^{(p)} - t_0^{(p)}}{2} \sum_{k=1}^{N} \sum_{i=0}^{N} w_k D_{k,i} \hat{\mathbf{x}}_i = \mathbf{0}_n, \quad p = 1, \dots, P \\
& \mathbf{e}^{L,(p)} \leq \mathbf{e}^{(p)}(\hat{\mathbf{x}}_0^{(p)}, \hat{\mathbf{x}}_N^{(p)}, t_0^{(p)}, t_f^{(p)}) \leq \mathbf{e}^{U,(p)}, \quad p = 1, \dots, P \\
& \mathbf{h}^{L,(p)} \leq \mathbf{h}^{(p)}(\hat{\mathbf{x}}_k^{(p)}, \hat{\mathbf{u}}_k^{(p)}, \tau_k) \leq \mathbf{h}^{U,(p)}, \quad k = 1, \dots, N, \quad p = 1, \dots P \\
& \Phi^{L,(p,q)} \leq \Phi^{(p,q)}(\hat{\mathbf{x}}_0^{(p)}, \hat{\mathbf{u}}_0^{(p)}, \hat{\mathbf{x}}_N^{(q)}, \hat{\mathbf{u}}_N^{(q)}) \leq \Phi^{U,(p,q)}, \quad p, q = 1, \dots, P
\end{aligned} \tag{S27}$$

To perform the discretization described in this subsection, we use the open-source C++ PSOC package $\mathcal{PSOPT}$[9].

Next we show that Eq. (S27) can be expressed in the typical NLP form[6]. Let $\mathbf{z}^{(p)}$ contain all of the variables for phase p.

$$\mathbf{z}^{(p)} = \begin{bmatrix} \hat{\mathbf{x}}_0^{(p)} \\ \vdots \\ \hat{\mathbf{x}}_N^{(p)} \\ \hat{\mathbf{u}}_0^{(p)} \\ \vdots \\ \hat{\mathbf{u}}_N^{(p)} \end{bmatrix} \in \mathbb{R}^{(n+m)} \tag{S28}$$

Next, let $\mathbf{z}$ contain the variables for every phase,

$$\mathbf{z} = \begin{bmatrix} \mathbf{z}^{(1)} \\ \vdots \\ \mathbf{z}^{(P)} \end{bmatrix} \in \mathbb{R}^{(N+1)(n+m)} \tag{S29}$$

With some algebraic manipulation, the entire discretized multi-phase OCP can be rewritten as an NLP in the typical form.

$$\begin{aligned}
\min_{\mathbf{z}} \quad & c(\mathbf{z}) \\
\text{s.t.} \quad & \mathbf{g}(\mathbf{z}) = \mathbf{0} \\
& \mathbf{d}(\mathbf{z}) \leq \mathbf{0}
\end{aligned} \tag{S30}$$

To solve the large-scale NLP in Eq. (S30) we employ an interior-point algorithm[6]. Specific details of the algorithm are outside the scope of this paper. We used the open-source C++ package Ipopt[10] to solve each instance of Eq. (S30). We direct interested readers who would like to learn more about the technical detailed involved when solving Eq. (S30) to the documentation provided with Ipopt.

The optimal solution returned, $\mathbf{z}^*$, is separated into its component parts; first by splitting it into the phases $\mathbf{z}^{(p)*}$, and second by reconstructing the discrete states and contrlol inputs, $\hat{\mathbf{x}}_i^*$ and $\hat{\mathbf{u}}_i^*$. The continuous time control inputs and states are then reconstructed using the Lagrange interpolating polynomials in Eq. (S19). With the continuous time states and control inputs, $\mathbf{x}^*(t)$ and $\mathbf{u}^*(t)$, we then verify that the necessary conditions are met to within an acceptable tolerance.

Supplementary Note

The Response of AVs to Constant Perturbation by Dual Therapies

Before solving the optimal control problem presented in the main text, we explore the capabilities of the dual therapies in terms of upregulate and downregulate with constant drug concentration as we did in Fig. 3 of the main manuscript. There, we plotted the long-time response of the system to an individual time-constant drug concentration (w) perturbation for the two sets of parameters $C_{\text{Nu}} = C_{\text{En}} = 0.1$ and $C_{\text{Nu}} = C_{\text{En}} = 0.6$. Similarly, in Fig. S3 and S4, we plot the long-time system AV response for the case of dual therapies with time-constant drug concentration perturbations.

In Fig. S3, we set the parameters $C_{\text{Nu}} = C_{\text{En}} = 0.1$. For these parameter values, in the absence of any drugs (control inputs), the sole attractor of the dynamical system corresponds to a high AV count (≈ 37). Fig. S3 shows the long-time AV response when the system is perturbed by different combinations of constant inputs. Note that those subsets that contain either drug 2 or 6 are capable of driving the AVs to zero if $w^{\max}$ is made large enough (pairs $\{2,3\}$, $\{2,4\}$, $\{2,6\}$, $\{3,6\}$, $\{4,6\}$, and $\{1,6\}$). For each pair $\{i,j\}$, we set $w_i = w_j$ and all other values $w_k = 0$, $k \neq i$ and $k \neq j$. The pair $\{3,4\}$ on the other hand is only capable of driving the AVs to ≈ 10 where any increase of $w^{\max}$ afterwards can produce no further results. Also, dual therapy $\{1,5\}$ is incapable of downregulate .

In Fig. S4, we set the parameters $C_{\text{Nu}} = C_{\text{En}} = 0.6$, for which the free evolution of the system is periodic (see Fig. 2 in the main text), and show the same long-time AV response results under constant drug concentration perturbation. For all dual therapies shown, small drug concentrations are unable to remove the oscillations present (denoted by the shaded regions). Similar to Fig. S3, we see that all drug combinations that contain either drug 2 or 6 are capable of driving the level of AVs to zero for $w^{\max}$ set large enough. Also, dual therapy $\{3,4\}$, as before, is only able to reduce the AVs level to ≈ 10 while the dual therapy $\{1,5\}$ instead upregulates the AVs.

Exhaustive Analysis of Two-Drug Combinations

In this section, we present simulation results for all possible dual therapies . First, we set both the parameters $C_{\text{Nu}} = C_{\text{En}} = 0.1$ for which the number of AVs at steady state in the absence of control inputs is equal to ≈ 37. We attempt to downregulate the number of AVs using pairs of drugs from the set $\{2,3,4,6\}$ so that there are a total of $\binom{4}{2} = 6$ combinations. A pair of drugs drawn from this set is called a dual therapy . If $\{i,j\}$ is a dual therapy , then we say $\{i\}$ and $\{j\}$ are its component monotherapies .

The goal is to investigate our ability to downregulate the number of AVs from the steady state value ≈ 37 to a lower value in a specified control time interval $[0, t_0]$ and, subsequently, to maintain the number of AVs near the target level for a second time interval $[t_0, t_f]$, by using each different dual therapy . We say a dual therapy is *viable* if it is capable of performing the goal stated. A dual therapy is deemed efficient if;

- the dual therapy is viable while at least one of its component monotherapies is not, and
- the total amount of drugs provided by the dual therapy is less than either of the component monotherapies .

To compare the efficiencies of the dual therapies we define $r^*_{i,k}(t) = \int_0^t u^*_i(\tau)d\tau$ as the total amount of drug i administered at time t as part of a $k =$ dual or $k =$ mono and introduce the quantities ρ_i and τ_i.

$$0 \leq \rho_i = \frac{r^*_{i,\text{dual}}(t_f)}{r^*_{i,\text{mono}}(t_f)} \leq 1, \tag{S31}$$

Note that $r^*_{i,\text{dual}}(t_f) \leq r^*_{i,\text{mono}}(t_f)$, as otherwise the solution of the dual therapy optimal control problem would be suboptimal with respect to the case that only drug i is used. We also define the ratio

$$\tau_i = \frac{\bar{t}_{i,\text{dual}} - \bar{t}_{i,\text{mono}}}{\bar{t}_{i,\text{mono}}} \tag{S32}$$

where $\bar{t}_{i,\text{dual}}$ is the time when drug i is activated (that is, the earliest time at which the drug injection rate is nonzero) as a part of a dual therapy and $\bar{t}_{i,\text{mono}}$ is the time when drug i is activated as a monotherapy . Note that $\tau_i > 0$ ($\tau_i < 0$) indicates a later (earlier) activation time of drug i as a part of dual therapy compared to as a monotherapy .

For our simulations, we set the upper bound of the drug concentrations to $w_i^{\max} = 2$ for each drug i, the time at which we apply the upper bound to the AVs to $t_0 = 120$ minutes, the time at which we end the simulation to $t_f = 240$ minutes, and we set the initial condition $\mathbf{x}(0)$ to be equal to the steady state solution of the system in the absence of control inputs with parameters $C_{\text{En}} = C_{\text{Nu}} = 0.1$. In Fig. S5, we plot the total drug administered $r_i(t) = \int_0^t u_i(\tau)d\tau$ in the interval $[0, t_f]$. The

plots on the diagonal panels, labeled (u_i, u_i), correspond to the monotherapies and the plots on the upper triangular panels, labeled (u_i, u_j), correspond to the dual therapies . Symmetric to each upper triangular panel (u_i, u_j), the corresponding lower triangular panel (u_j, u_i) contains the values of the ratios ρ_i and τ_i in Eqs. (S31) and (S32), respectively.

We notice from Fig. 3*A* in the main text that the only monotherapies which can downregulate the number of AVs from ≈ 37 to ≈ 10, with $w_i \leq 2$, is $\{4\}$. Thus, the red crosses in panels (u_2, u_2), (u_3, u_3) and (u_6, u_6) in Fig. S5 indicate that those monotherapies cannot solve the downregulate problem. Clearly, dual therapies $\{2, 4\}$, $\{3, 4\}$ and $\{4, 6\}$ are viable as drug $\{4\}$ as a monotherapy is viable. On the other hand, the dual therapies $\{2, 3\}$ and $\{3, 6\}$ are not viable. The most interesting dual therapy is $\{2, 6\}$ as neither component monotherapy is viable yet as a pair they are viable Thus by our stated goal and definitions, the dual therapy $\{2, 6\}$ is efficient according to our criteria. Also, dual therapy $\{3, 4\}$ is deemed efficient as the total consumption of drug 4 is much lower ($\rho_4 = 0.29$) than the total consumption of drug 4 as a monotherapy as shown in panel (u_3, u_4) in Fig. S5. We also observe the faster response of drug 4 as a part of the $\{3, 4\}$ dual therapy than its response as a monotherapy because $\tau_4 = 0.32 > 0$.

In Fig. S6, we consider the dual therapies by combining one of the downregulate drugs, 2, 3, 4 or 6, with one of the upregulate drugs, 1 or 5. A red cross in a panel again represents a monotherapy or a dual therapy that is not viable. While the dual therapies $\{1, 4\}$ and $\{4, 5\}$ are viable, they are not efficient as neither drugs 1 nor 5 are used (non-zero).

In Fig. S7, we present detailed results when we set the parameters $C_{\text{En}} = C_{\text{Nu}} = 0.6$, for which the dynamics in the absence of control inputs is oscillatory. In our numerical experiments, we attempt to downregulate the number of AVs from its initial periodic behavior to $x_5(t_0) \approx 10$ and to maintain the number of AVs near that value for the time interval $[t_0 = 120, t_f = 240]$. The red cross in panel (u_6, u_6) indicates the inability of drug 6 as a monotherapy to downregulate the AVs to the desired level. However, we found this drug to be particularly beneficial when used as a component in a dual therapy . We find that while all dual therapies are viable, the most efficient dual therapy is $\{2, 6\}$, as the total amount of drug 2 required is reduced by more than five folds when compared to the monotherapy $\{2\}$. A comparison with drug 6 alone is not possible as drug 6 as a monotherapy is not viable. The dual therapy $\{3, 6\}$ is also efficient by our definition, but only slightly as the amount of drug 3 used is hardly reduced, $\rho_3 = 0.96$. For all other dual therapies , one of the component drugs is never activated so while they may by viable, we do not consider them efficient.

In Fig. S9, we summarize the results when we attempt to upregulate the number of AVs to ≈ 37 in the same control time interval $[0, t_0]$ and, subsequently, maintain the number of AVs throughout the time interval $[t_0, t_f]$ by using dual therapy $\{1, 5\}$. We observe that, while the dual therapy $\{1, 5\}$ is viable, it is not efficient as drug 1 is never activated and so we must use the same amount of drug 5 as when it is used as a monotherapy .

In Fig. S10, we consider the dual therapies by combining one of the downregulate drugs, 2, 3, 4 or 6, with one of the upregulate drugs, 1 or 5. We observe that the dual therapies $\{1, 6\}$ and $\{5, 6\}$ are only efficient when $C_{\text{En}} = C_{\text{Nu}} = 0.6$. The other dual therapies while viable are not efficient as the upregulate component (either 1 or 5) is never activated (that is, non-zero).

Supplementary Tables

Supplementary Table S1. Parameters of the model (Eq. (1)). See "Formulation of the Model" in Supplementary Methods for discussion. The parameter values are dimensionless except as indicated.

Parameter	Value	Parameter	Value
$r_{b,12}$	0	k_1	1.00×10^{-1}
$r_{m,12}$	1.00×10^{1}	k_2	3.00×10^{-1}
θ_{12}	3.00×10^{-1}	k_3	4.00×10^{0}
n_{12}	4.00×10^{0}	k_4	1.00×10^{-1}
$r_{b,13}$	0	δ_1	3.10×10^{-4}
$r_{m,13}$	1.00×10^{1}	δ_2	1.93×10^{-3}
θ_{13}	6.00×10^{-1}	δ_3	5.78×10^{-3}
n_{13}	6.00×10^{0}	δ_4	1.15×10^{-2}
$r_{b,23}$	0	δ_5	2.31×10^{-3}
$r_{m,23}$	6.00×10^{0}	δ_6	1.16×10^{-3}
θ_{23}	1.00×10^{0}	r_b	0
n_{23}	4.00×10^{0}	r_m	1.00×10^{0}
$r_{b,21}$	1.00×10^{-1}	θ	5.00×10^{-1}
$r_{m,21}$	6.00×10^{0}	n	2.00×10^{0}
θ_{21}	6.00×10^{-1}	T	1.00×10^{0} (min)
n_{21}	4.00×10^{0}		
$r_{b,42}$	1.00×10^{-1}		
$r_{m,42}$	6.00×10^{0}		
θ_{42}	5.00×10^{-1}		
n_{42}	4.00×10^{0}		

Supplementary Table S2. Summary of measured drug half-lives used to set values for the drug clearance rate constants $\delta_1, \ldots, \delta_6$ in Eq. (1). Each half-life, $t_{1/2,i}$, is the measured half-life of a representative of drug type i. See the references cited in the table for details about the drugs and measurements.

Drug i	Half-life $t_{1/2,i}$	Value (h^{-1})	Rate constant δ_i	Value (min^{-1})	Reference
1	$t_{1/2,1}$	~ 37	δ_1	3.10×10^{-4}	Sato et al.[11]
2	$t_{1/2,2}$	~ 6	δ_2	1.93×10^{-3}	Baselga et al.[12]
3	$t_{1/2,3}$	~ 2	δ_3	5.78×10^{-3}	Milkiewicz et al.[13]
4	$t_{1/2,4}$	~ 1	δ_4	1.15×10^{-2}	Engers et al.[14]
5	$t_{1/2,5}$	~ 5	δ_5	2.31×10^{-3}	Cameron et al.[15]
6	$t_{1/2,6}$	~ 10	δ_6	1.16×10^{-3}	Juric et al.[16]

Supplementary Figures

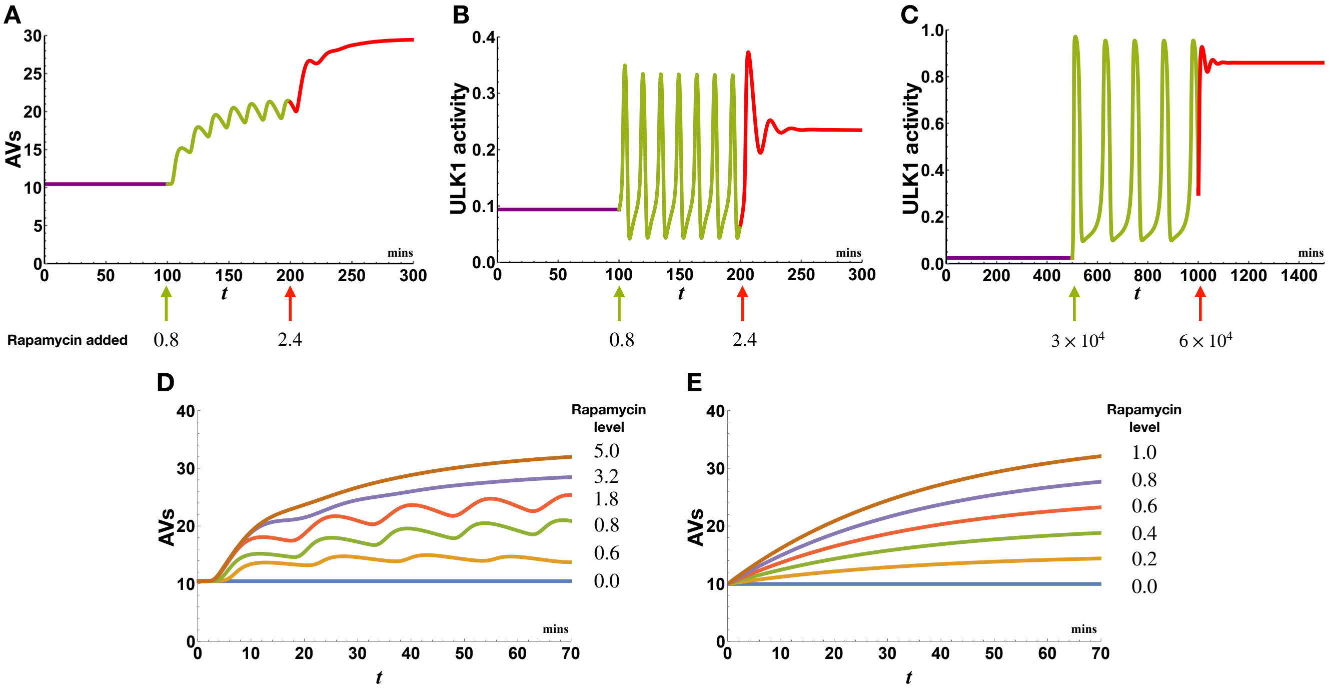

Supplementary Fig. S1. Comparison of simulations based on Eq. (1) and simulations based on models of Szymańska et al.[1] (Ref. 33 in the main text) and Martin et al.[2] (Ref. 34 in the main text). (*A*) AV dynamics, $x_5(t)$, predicted by Eq. (1). The value of x_5 is initially steady and low; the system is perturbed by two additions of rapamycin at time $t = 100$ and 200 min, as indicated. (*B*) Dynamics of ULK1 activity, $x_2(t)$, predicted by Eq. (1). The conditions considered are the same as those in panel *A*. (*C*) Dynamics of ULK1 activity predicted by the model of Szymańska et al.[1]. The conditions considered here correspond qualitatively to those considered in panels *A* and *B*. Initially, there is no rapamycin. Later, a low dose of rapamycin is added. Still later, a high dose of rapamycin is added. Note that the models of Eq. (1) and Szymańska et al.[1] have different timescales. This situation is partly a consequence of requiring Eq. (1) to reproduce the AV dynamics measured by Martin et al.[2]. Szymańska et al.[1] showed that the qualitative pattern of behavior illustrated here is a robust feature of known regulatory interactions among AMPK, MTORC1, and ULK1 (i.e., the pattern of behavior is insensitive to parameter variations). Furthermore, it should be noted that the model of Szymańska et al.[1] does not track AVs. Thus, there is no direct comparison to be made with the time course shown in panel *A*. (*D*) AV dynamics predicted by Eq. (1). AV production is stimulated by the addition of rapamycin at the (dimensionless) doses indicated in the legend. (*E*) AV dynamics predicted by the model of Martin et al.[2]. As in panel *D*, autophagy is induced by the addition of rapamycin at different doses, as indicated in the legend. For further discussion, see "Formulation of the Model" in Supplementary Methods.

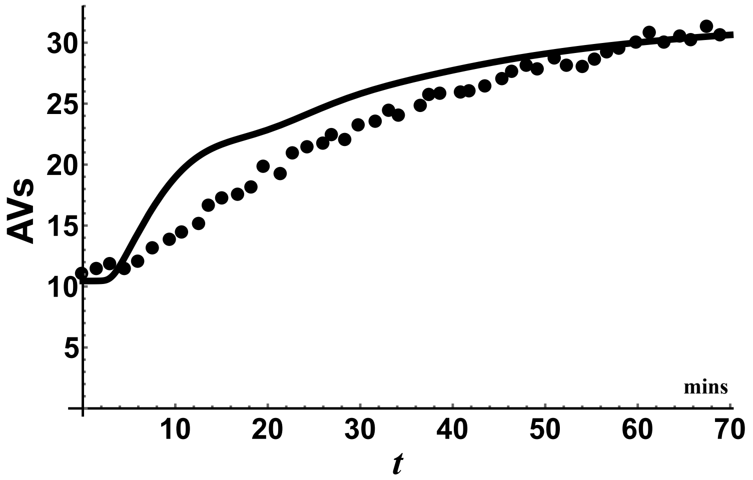

Supplementary Fig. S2. Comparison of simulations based on Eq. (1) and data generated by Martin *et al.*[2] (Ref. 34 in the main text). We parameterized the model of Eq. (1) to roughly reproduce autophagic vesicle (AV) population dynamics reported by Martin et al.[2]. Our goal was not to reproduce the observed dynamics exactly but rather to select parameters that yield induction dynamics on a comparable timescale and a comparable maximal range of regulation. The measured dynamics were induced by inhibition of MTORC1 using AZD8055, a catalytic MTOR inhibitor. Dynamics were similar when autophagy was induced using rapamycin[2]. The curve corresponds to a simulation based on Eq. (1). Each dot corresponds to the average of AV counts measured in a series of fluorescence microscopy experiments[2]. The data shown here are taken from Figure 6B in Martin et al.[2]. For further discussion, see “Formulation of the Model” in Supplementary Methods.

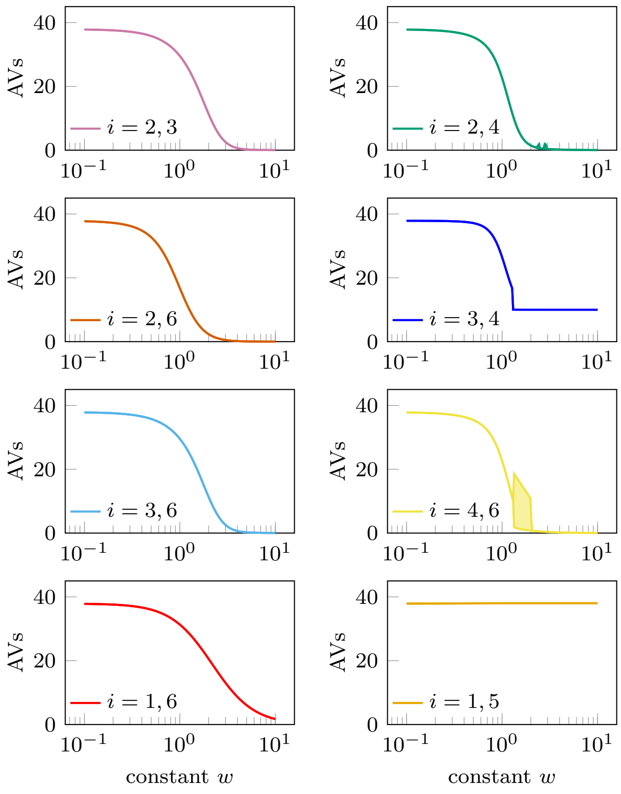

Supplementary Fig. S3. The dual therapy long-time response of the system in the case of time-constant drug concentration perturbations for the parameters $C_{\text{Nu}} = C_{\text{En}} = 0.1$. Note that when w is small, the system is oscillatory (represented by the shaded region in the panels). For each pair of drug, there is some value of w required to overcome the natural oscillatory behavior of the system.

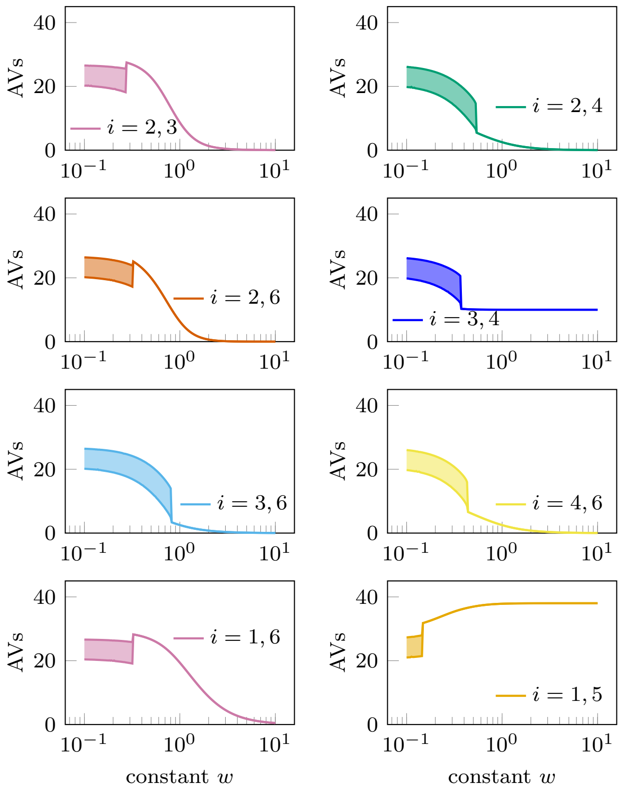

Supplementary Fig. S4. The dual therapy long-time response of the system in the case of time-constant drug concentration perturbations for the parameters $C_{\text{Nu}} = C_{\text{En}} = 0.6$.

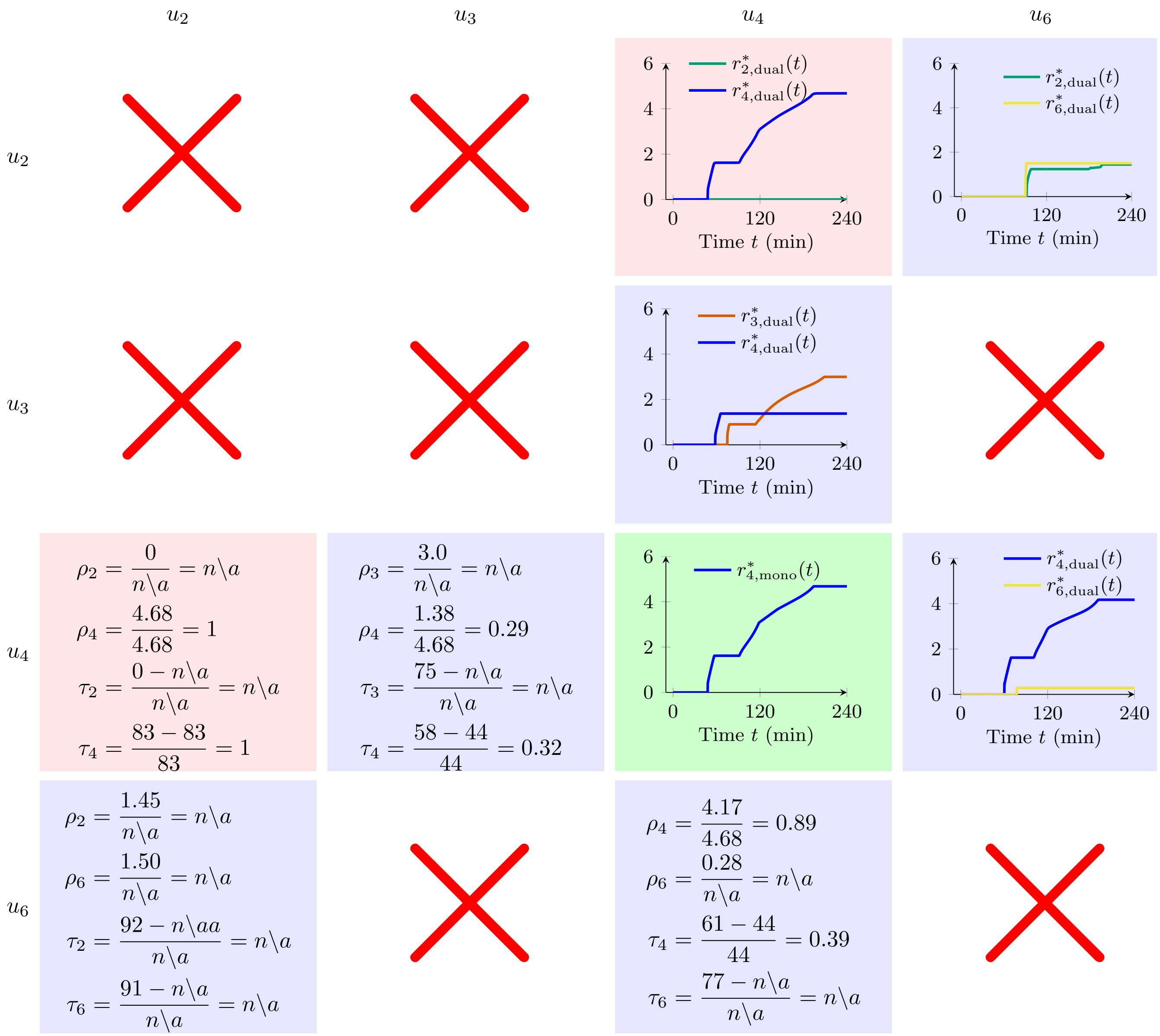

Supplementary Fig. S5. The parameter set $C_{\mathrm{Nu}} = C_{\mathrm{En}} = 0.1$. The target level of AVs is set $x_5^f = 10$ and the maximum drug concentration is set $w_i^{\max} = 2$. The diagonal panels represent monotherapies while off-diagonal panels represent dual therapies . Super-diagonal panels plot the total drug administered and sub-diagonal panels show the efficiency ratios described in the text of the dual therapies . Those diagonal panels with a red cross correspond to those monotherapies which are not viable. The only viable monotherapy is $\{4\}$, which is shown with a green background. The off-diagonal panel with a red background for dual therapy $\{2, 4\}$ is viable, but it is not efficient as drug 2 is not activated. The other three viable dual therapies , $\{2, 6\}$, $\{3, 4\}$, and $\{4, 6\}$ are both viable and efficient, shown with a blue background.

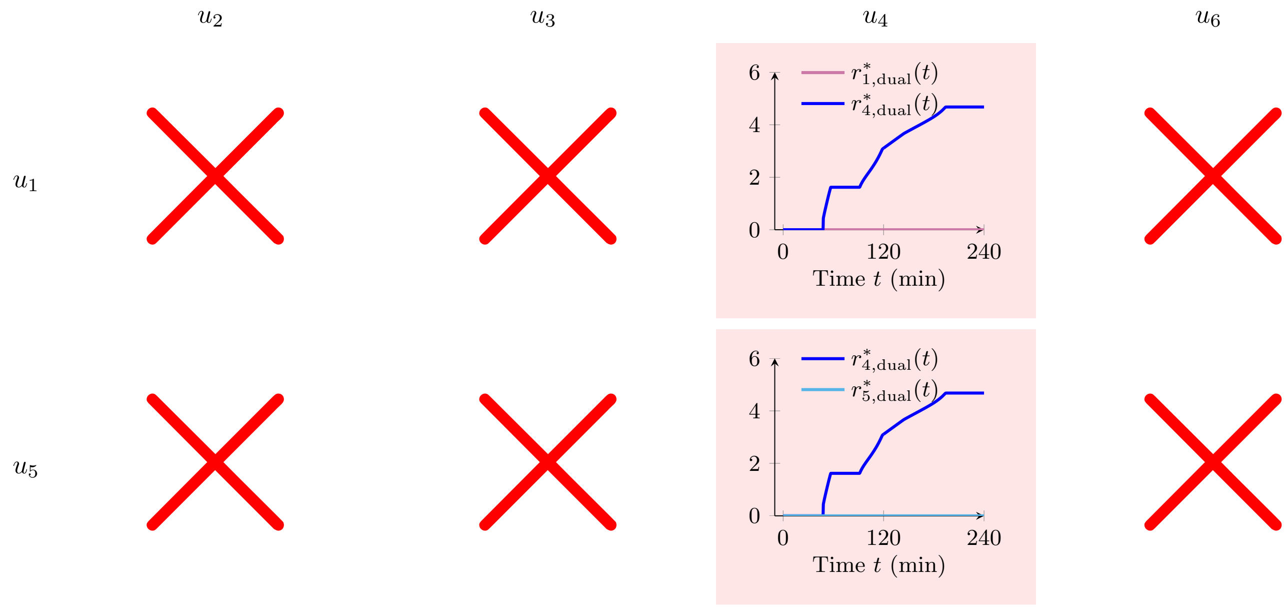

Supplementary Fig. S6. The parameter set $C_{\text{Nu}} = C_{\text{En}} = 0.1$. The target level of the AVs is set to $x_5^f = 10$ and the maximum drug concentration is set to $w_i^{\max} = 2$. Here we consider those dual therapies which combine one downregulate drug (2, 3, 4, or 6) with one of the upregulate drugs (1 or 5). Most of the dual therapies are not viable, which is represented with a red cross. The two viable dual therapies , $\{1, 4\}$ and $\{4, 5\}$, are not viable and so they are shown with a red background.

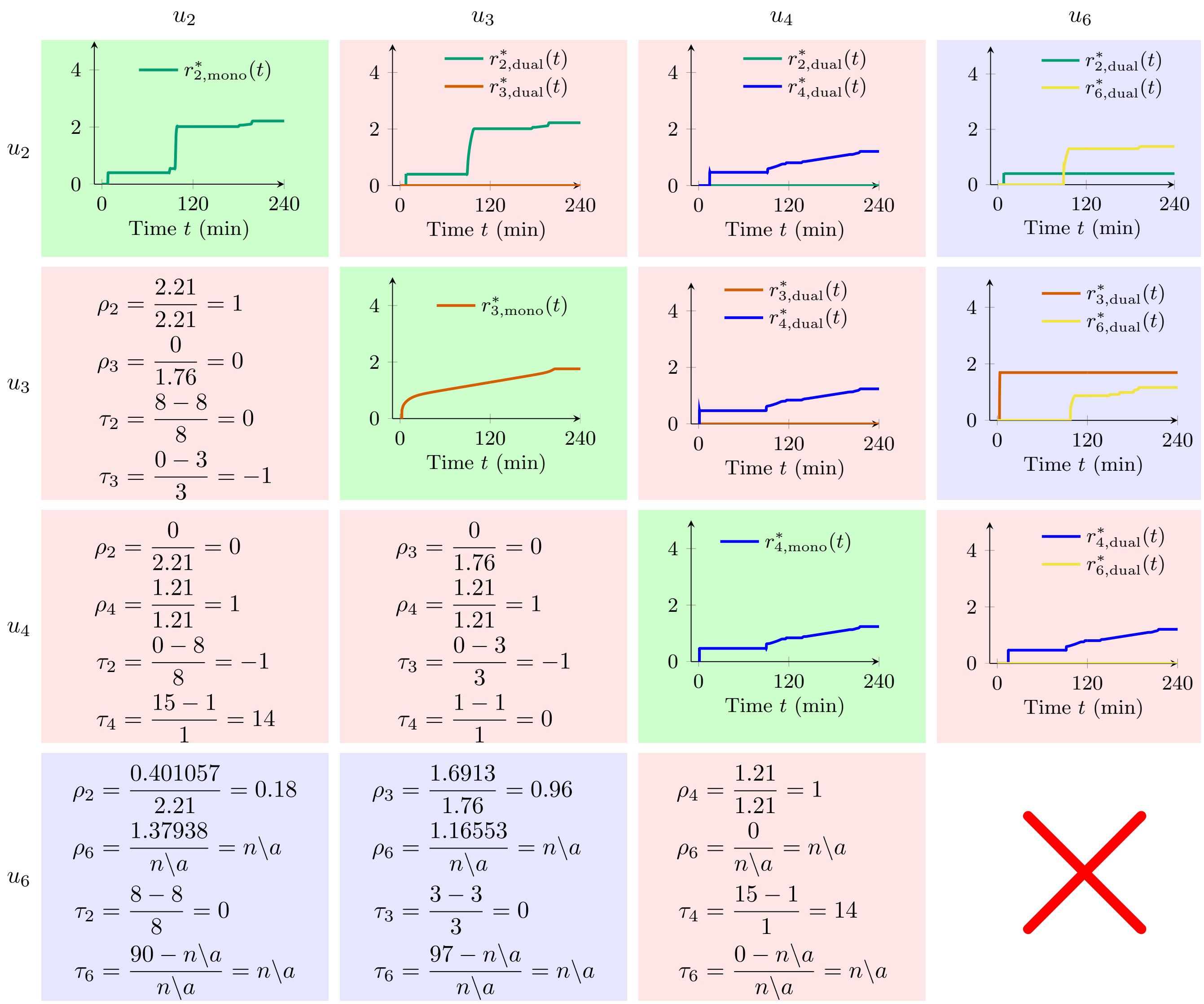

Supplementary Fig. S7. The parameter set $C_{\text{Nu}} = C_{\text{En}} = 0.6$. The target level of the AVs is set to $x_5^f = 10$ and the maximum drug concentration is set to $w_i^{\max} = 2$. The diagonal panels (u_i, u_i) (with a green background) show the total drug administered for monotherapies . The red cross on the diagonal panel corresponding to monotherapy $\{6\}$ represents the fact $\{6\}$ is not viable. The upper triangular panels (u_i, u_j), $i < j$, show the total drugs administered for dual therapies . In the lower triangular panels (u_j, u_i), $i < j$, we compare the dual therapies to their component monotherapies with the efficiency parameters τ and ρ. A red background in an off-diagonal panel represents those dual therapies which are viable but not efficient with respect to its component monotherapies . A blue background represents those dual therapies which are both viable and efficient.

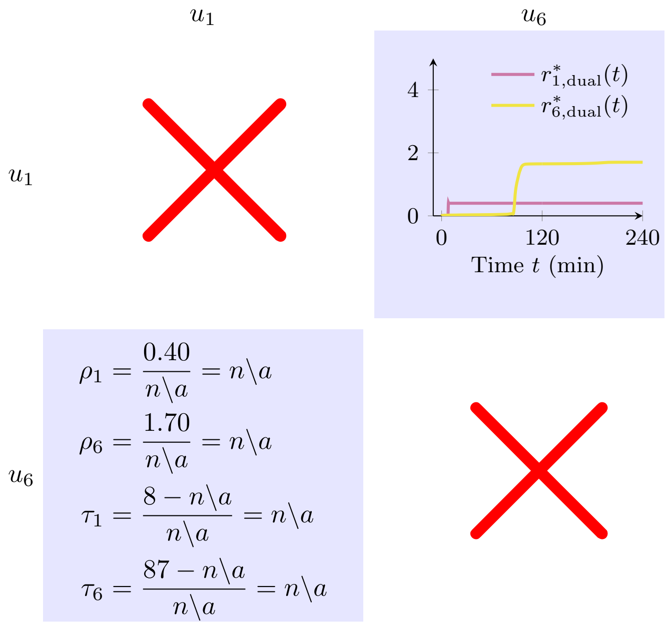

Supplementary Fig. S8. The parameter set $C_{\text{Nu}} = C_{\text{En}} = 0.6$. The target level of the AVs is set to $x_5^f = 10$ and the maximum drug concentration is set to $w_i^{\max} = 2$. The red crosses on the diagonal panels represents the fact that the monotherapies $\{1\}$ and $\{6\}$ are not viable. On the other hand, the dual therapy $\{1, 6\}$ is both viable and efficient. The viable dual therapies composed of two monotherapies which are not viable alone are the type of dual therapies we find most interesting as they are not obvious when analyzing the monotherapies alone. In the lower triangular panel we compare the dual therapy to its component monotherapies with respect to the efficiency ratios ρ and τ.

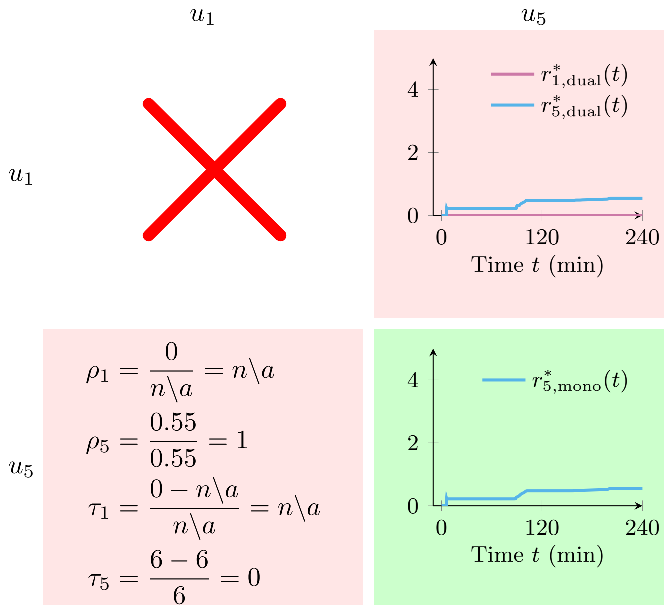

Supplementary Fig. S9. The parameter set $C_{\text{Nu}} = C_{\text{En}} = 0.6$. The target level of the AVs is set to $x_5^f = 10$ and the maximum drug concentration is set to $w_i^{\max} = 2$. The diagonal panels represent the monotherapies $\{1\}$ and $\{5\}$. A red cross on the diagonal panel for monotherapy $\{1\}$ represents the fact $\{1\}$ is not viable. On the other hand, monotherapy $\{5\}$ is viable (shown with a green background). The dual therapy $\{1, 5\}$ is viable (total drug administered is shown with the red background in the upper triangular panel) but is not efficient. The inefficiency is shown in the lower triangular panel with the efficiency ratios $\rho_5 = 1$.

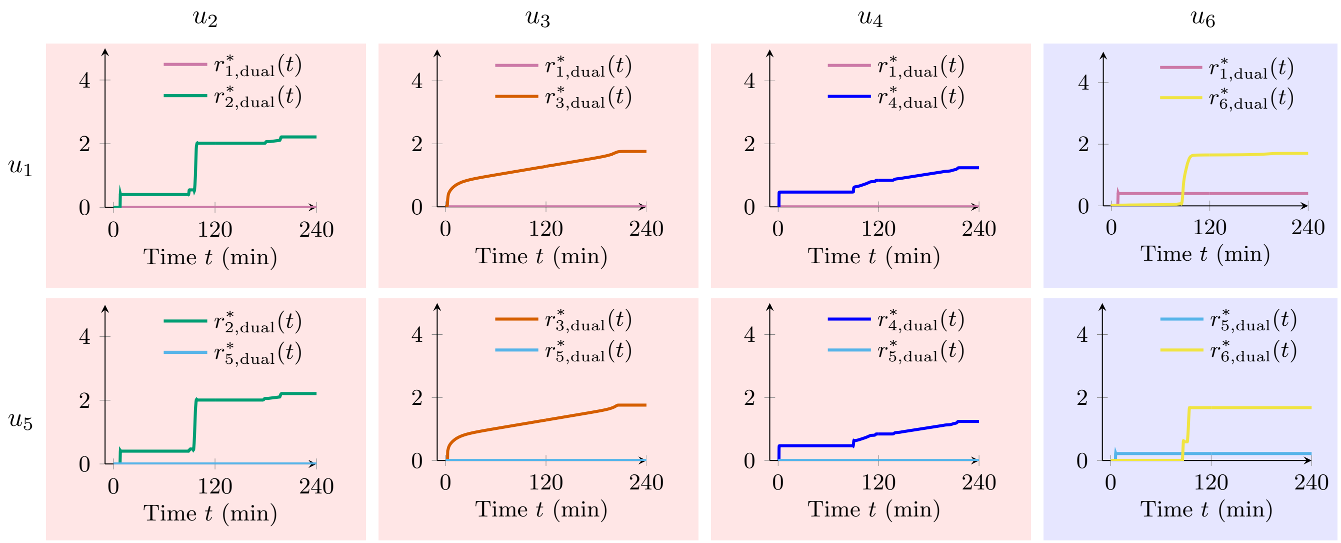

Supplementary Fig. S10. The parameter set $C_{\text{Nu}} = C_{\text{En}} = 0.6$. The target level of AVs is set to $x_5^f = 10$ and the maximum drug concentration is set to $w_i^{\max} = 2$. Here we consider those dual therapies compose of one downregulate drug (2, 3, 4, or 6), and one upregulate drug (1 or 5). Those panels with a red background represent dual therapies which are viable but not efficient while the two dual therapies $\{1, 6\}$ and $\{5, 6\}$ are efficient. In fact, as seen before, neither the component monotherapy $\{6\}$ nor the upregulate drugs are viable for this parameter set, so these efficient dual therapies are particularly interesting as they could not be found when analyzing the monotherapies alone.

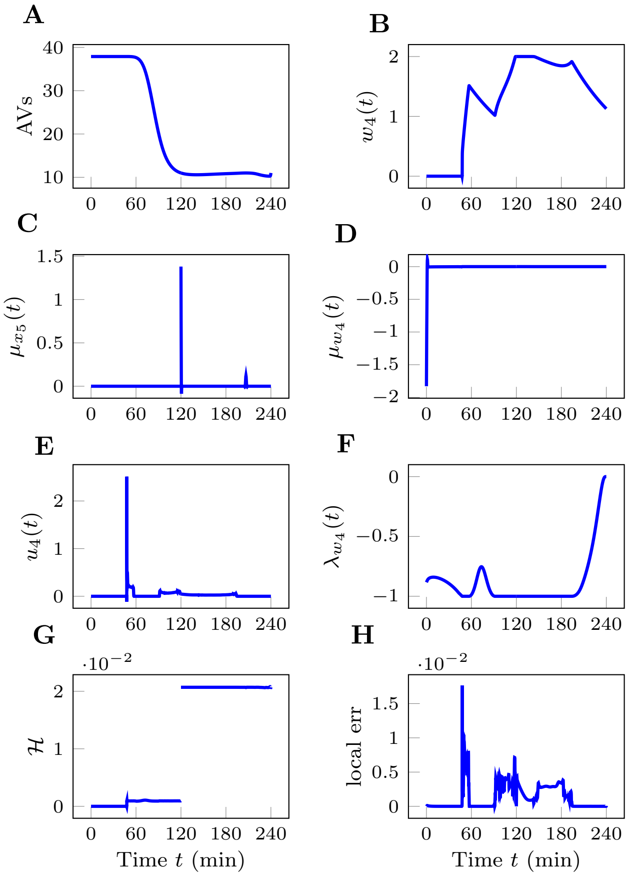

Supplementary Fig. S11. a) The optimal time evolution of the amount of AVs. b) The optimal time evolution of the drug concentration $w_4(t)$. c) The time evolution of the path covector μ_{x_5} associated with the upper bound applied to $x_5(t)$. d) The time evolution of the path covector μ_{w_4} associated with the state $w_4(t)$. e) The optimal time evolution of the drug $u_4(t)$. f) The costate $\lambda_{w_4}(t)$ associated with the state $w_4(t)$. g) The time evolution of the lower Hamiltonian $\mathcal{H}$. h) The relative local discretization error at each time t.

References Cited in Supplementary Information

1. Szymańska, P., Martin, K. R., MacKeigan, J. P., Hlavacek, W. S. & Lipniacki, T. Computational analysis of an autophagy/translation switch based on mutual inhibition of MTORC1 and ULK1. *PLoS One* **10**, e0116550, DOI: 10.1371/journal.pone.0116550 (2015).
2. Martin, K. R. *et al.* Computational model for autophagic vesicle dynamics in single cells. *Autophagy* **9**, 74–92, DOI: 10.4161/auto.22532 (2013).
3. Chylek, L. A. *et al.* Rule-based modeling: a computational approach for studying biomolecular site dynamics in cell signaling systems. *Wiley Interdiscip. Rev. Syst. Biol. Medicine* **6**, 13–36, DOI: 10.1002/wsbm.1245 (2014).
4. Faeder, J. R., Blinov, M. L. & Hlavacek, W. S. Rule-based modeling of biochemical systems with BioNetGen. *Methods Mol. Biol.* **500**, 113–167, DOI: 10.1007/978-1-59745-525-1_5 (2009).
5. Kirk, D. E. *Optimal Control Theory: an Introduction* (Courier Corporation, 2012).
6. Nocedal, J. & Wright, S. *Numerical Optimization* (Springer, 2006).
7. Ross, I. M. *A Primer on Pontryagin's Principle in Optimal Control* (Collegiate publishers, 2015).
8. Rao, A. V. A survey of numerical methods for optimal control. *Adv. Astronaut. Sci.* **135**, 497–528 (2009).
9. Becerra, V. M. Solving complex optimal control problems at no cost with PSOPT. In *Computer-Aided Control System Design (CACSD), 2010 IEEE International Symposium on*, 1391–1396, DOI: 10.1109/CACSD.2010.5612676 (IEEE, 2010).

10. Wächter, A. & Biegler, L. T. On the implementation of an interior-point filter line-search algorithm for large-scale nonlinear programming. *Math. Program.* **106**, 25–57, DOI: 10.1007/s10107-004-0559-y (2006).

11. Sato, E. *et al.* Temporal decline in sirolimus elimination immediately after pancreatic islet transplantation. *Drug Metab. Pharmacokinet.* **21**, 492–500, DOI: 10.2133/dmpk.21.492 (2006).

12. Baselga, J. *et al.* Buparlisib plus fulvestrant versus placebo plus fulvestrant in postmenopausal, hormone receptor-positive, HER2-negative, advanced breast cancer (belle-2): a randomised, double-blind, placebo-controlled, phase 3 trial. *The Lancet Oncol.* **18**, 904–916, DOI: 10.1016/S1470-2045(17)30376-5 (2017).

13. Milkiewicz, K. L. *et al.* Improvement in oral bioavailability of 2,4-diaminopyrimidine c-Met inhibitors by incorporation of a 3-amidobenzazepin-2-one group. *Bioorganic & Medicinal Chem.* **19**, 6274–6284, DOI: 10.1016/j.bmc.2011.09.006 (2011).

14. Engers, D. W., Frist, A. Y., Lindsley, C. W., Hong, C. C. & Hopkins, C. R. Synthesis and structure–activity relationships of a novel and selective bone morphogenetic protein receptor (BMP) inhibitor derived from the pyrazolo [1.5-a] pyrimidine scaffold of dorsomorphin: the discovery of ML347 as an ALK2 versus ALK3 selective MLPCN probe. *Bioorganic & Medicinal Chem. Lett.* **23**, 3248–3252, DOI: 10.1016/j.bmcl.2013.03.113 (2013).

15. Cameron, K. O. *et al.* Discovery and preclinical characterization of 6-chloro-5-[4-(1-hydroxycyclobutyl) phenyl]-1 H-indole-3-carboxylic acid (PF-06409577), a direct activator of adenosine monophosphate-activated protein kinase (AMPK), for the potential treatment of diabetic nephropathy. *J. Medicinal Chem.* **59**, 8068–8081, DOI: 10.1021/acs.jmedchem.6b00866 (2016).

16. Juric, D. *et al.* A first-in-human, phase I, dose-escalation study of TAK-117, a selective PI3Kα isoform inhibitor, in patients with advanced solid malignancies. *Clin. Cancer Res.* DOI: 10.1158/1078-0432.CCR-16-2888 (2017).
